# Public sector reforms and their impact on the level of corruption: A systematic review

**DOI:** 10.1002/cl2.1173

**Published:** 2021-05-24

**Authors:** Giulia Mugellini, Sara Della Bella, Marco Colagrossi, Giang Ly Isenring, Martin Killias

**Affiliations:** ^1^ Università della Svizzera Italiana (USI) and University of St. Gallen (HSG) in 2018, during the development of the systematic review. Via G. Buffi 13 Lugano Switzerland; ^2^ Scuola Universitaria Professionale della Svizzera Italiana (SUPSI) and University of St. Gallen (HSG) in 2018, during the development of the systematic review. Via Cantonale 16e Manno Switzerland; ^3^ European Commission, DG JRC, Directorate I – Competences, Unit I.1 ‐ Monitoring, Indicators & Impact Evaluation Ispra VA Italy; ^4^ Crime and Criminal Justice Section, Swiss Federal Statistical Office, Espace de l'Europe 10 Neuchâtel Switzerland; ^5^ Universität St. Gallen, Law School. Tigerbergstrasse 21 St.Gallen CH Switzerland; ^6^ Killias Research & Consulting Rathausgasse 30, P.O. 2094 Lenzburg 2 Switzerland

## Abstract

**Background:**

In spite of the large number of anti‐corruption reforms implemented in different countries, there has been little research that empirically and systematically assesses the impact of these efforts.

**Objectives:**

The main objective of this review is to identify what works in curbing corruption in the public sector, by meta‐analyzing the findings of published and unpublished evaluations of different types of anti‐corruption interventions in different countries. The focus of this review is administrative corruption, namely corrupt acts involving civil servants in their dealings with their superiors, during the implementation of public policies, or while interacting with the public for service delivery. Political corruption (in the adoption of laws, regulations, and policies), and private‐to‐private corruption (involving only private actors) are excluded from this review.

**Search methods:**

The literature search was conducted by querying three widely recognized electronic databases: RePEc, SSRN, and Web of Science. These databases are considered the most comprehensive in the socio‐economic field of research. The main grey literature repositories were also queried. Both published and unpublished studies were searched on the basis of specific combinations of keywords. The terms used to define queries were based on the “types of corruption”, “types of interventions/policies/reforms” and “study design” search strings. Specific conventions were used to “explode” or “truncate” keywords as appropriate. Screening of the references (i.e., snowballing) of the identified studies was also performed, and a reverse snowballing approach on Google Scholar was used. In order to ensure replicability, all searches were stored into Covidence, an online software developed by the Cochrane community for screening studies and extracting data for systematic reviews.

**Selection criteria:**

Any study that included experimental evaluations (randomized controlled trials) of interventions developed for use in the public sector (e.g., public administration, education, health, etc.) to curb administrative corruption has been included in this review without any geographical or temporal limitations. Only studies written in or translated into English have been considered.

**Data collection and analysis:**

Two review authors read the titles and abstracts of identified studies in order to determine their eligibility against the inclusion/exclusion criteria. When a title or abstract could not be included or rejected with certainty, the full text of the article was reviewed. In case of disagreement about whether or not a study should be included, the lead author (Giulia Mugellini), together with Martin Killias acted as arbitrator. The relevant information from identified studies was extracted independently by two review authors, following the guidelines of the Campbell Collaboration. The studies were assessed using the Cochrane Risk of Bias checklist as a basis.

The effect size selected for the analysis was the Fisher's z‐score transformation of the partial correlation coefficient. For the meta‐analysis, random effect(s) models were estimated. Meta‐regression analysis models were then used to investigate the determinants behind the observed between‐ and within‐study heterogeneity. Ten different covariates were included in the meta‐regression models in order to control for the type of intervention, the type of corruption, the level of national income, the quality of the study and the type of participants involved in laboratory experiments.

**Results:**

The initial literature search led to the identification of 70 studies. Approximately one‐third of the studies were excluded at the title/abstract stage because they either did not evaluate any anti‐corruption intervention but simply assessed the relationship between corruption and other phenomena, or because the study design was not based on randomized controlled trials. Another 14 studies were excluded only after a full‐text assessment. At this stage, the main reasons for exclusion were related to an unsuitable type of corruption (e.g., when the focus of the paper was political corruption, or private‐to‐private corruption instead of administrative corruption), the lack of regression output, or an unsuitable study design. At the end of the selection process, 29 studies resulted as eligible for inclusion.

All the selected studies were written in English. The publication years ranged from 2007 to 2018. The majority of the selected studies (20) investigates the effect of anti‐corruption interventions in high‐ and upper‐middle income countries (Austria, Brazil, Canada, China, Germany, Italy, Mexico, the Netherlands, Thailand, the United Kingdom, and the United States). Nine studies focused on low‐ and low‐middle income countries (Burkina Faso, Burundi, Ethiopia, India, Indonesia, Pakistan, Tanzania, and Uganda). All of them were randomized experiments. Twenty‐five of these experiments were conducted in a laboratory, while four of them were field experiments.

As to the type of outcome, the majority (18) of the selected studies addressed bribery (either active or passive), while 11 studies considered misappropriation of public resources (embezzlement). In terms of anti‐corruption interventions, 19 studies tested the effect of deterrence interventions, while 10 studies focused on policies based on organizational and cultural change.

Overall, the meta‐analysis’ findings indicate that the identified interventions decrease the level of corruption. Results are statistically significant (p < 0.01) and robust to different heterogeneity estimators—that is, (restricted) maximum likelihood and method of moment estimators. The observed high level of heterogeneity—$I^{2}$ is equal to 92.36%, of which 43.78% is due to between‐study heterogeneity and 48.57% to within‐study heterogeneity—albeit in line with other meta‐analyses in economics, suggests the need for meta‐regression analyses.

To investigate the determinants behind the between‐ and within‐study heterogeneity of the observed effect, both a random effect model and a multilevel model were adopted.

The results of the multilevel model show that:

1) Control and deterrence interventions are more effective than organizational and cultural reforms in reducing corruption in the public sector.
2) Combining different interventions reduces corruption more than single interventions.
3) Interventions are more effective in preventing misappropriation of public resources (embezzlement) than passive or active bribery.

Finally, the Funnel Asymmetry Test (FAT), conducted with both additive and multiplicative dispersion terms, shows no evidence of a strong publication bias in the literature.

**Authors’ conclusions:**

The results of this systematic review, based on a combination of laboratory and field experiments, demonstrate that increasing the expected monetary costs (e.g., sanctions) of corruption or the probability of detection (e.g., audit risk) is more effective than organizational, cultural and educational interventions in curbing administrative corruption, at least in the short term. However, this result might be due to the fact that the majority of selected studies are based on lab‐experiments, where the assessment of the intervention is almost concurrent to its development. Short‐term evaluations might fail to identify the effect of organizational and cultural interventions. Indeed, these interventions are based on structural changes in the organization of the system and the ethical and cultural education of public officials and might, thus, entail long periods to display their results on the level of corruption. Nevertheless, a combination of different interventions proves to be more effective than single interventions. For example, policies guaranteeing impunity to officials or citizens who report corrupt practices (principal witness/leniency treatment) are more effective if associated with a high probability of audit than leniency alone. A low probability of detection can be compensated by the threat of high fines in reducing both the amount and the likelihood of bribe demands. To the contrary, a high probability of detection had no effect in the absence of severe sanction threats.

The importance of the organizational and cultural environment in which the intervention is implemented clearly emerged in the literature. When possible, the characteristics of the settings where the interventions were developed were included in the meta‐regression analysis (such as the level of income of the countries). When it was not possible to measure contextual factors and their interaction with the main intervention, a qualitative analysis was performed to reveal the complexities of these interactions.

This additional analysis shows that the impact of the interventions was found to be affected by the likelihood of the continued interactions between bribe takers and givers, the amount and probability of fines, and the size of the bribe, among others. For example, reporting mechanisms and leniency policies increase their potential in combination with interventions that limit agent's exposure to one another – such as staff rotation. The success of audit risk on corruption is strongly dependent on the seriousness of the potential sanction and the probability that a sanction is applied. Some differences also emerge between high‐ and low‐corruption countries regarding the effectiveness of anti‐corruption interventions. For example, measures tending to increase social blame of corrupt practices work in low‐corruption countries. Adding punishments in environments where actors’ behavior is tightly monitored increases compliance, but more so in environments where corruption is the exception rather than the rule.

In terms of implications for research, the fact that control and deterrence turns out to be more effective than organizational and cultural interventions in curbing administrative corruption confirms the importance of economic theories (and cost‐benefit analysis). However, the meta‐analysis also demonstrates the effectiveness of combining different types of interventions.

This is true not only when combining policies reinforcing control and deterrence (monitoring frequency, detection probability and amount of fines), but also when policies based on organizational and cultural change are added (e.g., staff rotation and leniency). In particular, the role of moral levers in preventing corruption emerges, and especially the importance of strengthening professional identity and values in order to avoid conflicts between an individual's private interests and his/her public role. These results highlight the importance of going beyond economic models for explaining corruption, and considering the moral and cultural mechanisms underlying this phenomenon.

It also emerges the need to understand how different forms of corruption operate in practice at macro‐ (cross‐country), meso‐ (country/nation‐state) and micro‐ (individual) level. In particular, individual‐level factors, such as the strive for power, low self‐control, loss aversion and risk acceptance would need to be addressed.

It would be interesting to distinguish, when more experimental studies will be available, between top‐down (from supervisors to officials) and bottom‐up (from citizens to officials) interventions.

From a methodological point of view, it could be tested whether the results change according to the types of games used as a basis for the corruption experiments (e.g., behavioral game theory, trust game, etc.) and according to the setting in which the experiment was conducted (e.g., context‐free versus in‐context presentation of experimental tasks).

Considering the effect of sensitization messages in reducing bribery demand, we would encourage researchers to develop other corruption experiments that explore the impact of interventions in fostering professional self‐identity, as well as the impact of organizational family culture on corruption. Furthermore, this review highlights the need for a comprehensive classification of anti‐corruption policies that distinguishes interventions by type of corruption, risk factors, type of policy tool and administrative sector.

## 1 PLAIN LANGUAGE SUMMARY

### 1.1 Public sector corruption: control and deterrence is more effective than organizational and cultural reform

Control and deterrence interventions are more effective than organizational and cultural reforms in curbing corruption in the public sector, at least in the short term. The combination of different types of interventions is more effective than single interventions.

### 1.2 What is this review about?

This review addresses corruption in public administration. This includes corrupt acts involving civil servants in their dealings with their superiors, during the implementation of public policies, or while interacting with the public for service delivery. Administrative corruption is distinct from political corruption, and from private‐to‐private corruption.

This review covers any type of intervention developed in the public sector with the aim of deterring or preventing administrative corruption. Two main groups of interventions to curb administrative corruption are considered: 1) control and deterrence, and 2) organizational and cultural reforms.

**What is the aim of this review?**

This Campbell systematic review evaluates the impact on administrative corruption of public sector interventions based on control and deterrence, and on organizational and cultural change. The review synthetizes evidence from 29 high‐quality studies based on randomized controlled trials.

### 1.3 What studies are included in this review?

Twenty‐nine studies match the inclusion criteria. They span the period 2007 to 2018 and cover 16 different countries. All studies are randomized controlled trials, with 25 conducted in a laboratory, while four are field experiments.

### 1.4 What are the main findings of this review?

Do anti‐corruption interventions work in the public sector? Yes. Administrative corruption is reduced by control and deterrence interventions. But the reduction in corruption brought about by organizational and cultural interventions is not statistically significant. Anti‐corruption interventions can be more effective in reducing misappropriation of public resources than discouraging bribery.

The simultaneous combination of more than one intervention is more effective than single interventions. For example, policies guaranteeing impunity to officials or citizens who report corrupt practices (leniency treatment) are more effective if associated with a high probability of audit (detection), than leniency alone. A low probability of detection can be compensated by the threat of high fines in reducing both the amount and the likelihood of bribe demands. Conversely, a high probability of detection and low fines have no effect on either.

### 1.5 What do the findings of this review mean?

This review provides an in‐depth synthesis of the available evidence over 11 years and 16 countries. The findings suggest that policies based on control and deterrence are more effective in curbing corruption in the public sector than interventions based on organizational and cultural change.

The impact of the intervention may be affected by the likelihood of continued interactions between bribe takers and givers, and the prevalence of corruption. Measures tending to increase social blame of corrupt practices work in low‐corruption countries. In environments where corruption is the exception rather than the rule, adding punishments where actors’ behavior is tightly monitored increases compliance.

The fact that control and deterrence turn out to be more effective than organizational and cultural interventions in curbing administrative corruption confirms the importance of economic theories. However, combining different types of interventions works better than single measures. This is true not only when combining policies reinforcing control and deterrence, but also when policies based on organizational and cultural change are added.

In particular, the role of moral levers in preventing corruption emerges, and especially the importance of strengthening professional identity and values to avoid conflicts between an individual's private interests and public role. Moreover, bonuses and penalties may backfire because they lower the moral cost of corruption by diverting officials away from their sense of duty and their ethical responsibilities. Sensitization messages stressing public officials’ professional identity and position increase the moral cost of bribery.

These results highlight the importance of going beyond economic models for explaining corruption. Moral and cultural mechanisms remain important to understand how different forms of corruption emerge at macro (cross‐country), meso (country/nation‐state), and micro (individual) levels. In particular, individual‐level factors, such as the drive for power, low self‐control, loss aversion and risk acceptance need to be addressed.

### 1.6 How up‐to‐date is this review?

The review authors searched for studies up to 2018.

## 2 BACKGROUND

In the past decades, fighting corruption has emerged as a major component of reform programs in many countries, mainly as a reaction to the ratification of international convention (e.g., the OECD Anti‐Bribery Convention – 1997, and the UN Convention against Corruption ‐ 2004).According to Johnsøn and Søreide (2013: 1‐2), even if in the last two decades a substantial amount of empirical work has been done in order to understand the effects of anti‐corruption interventions in different countries, “producing evidence that these interventions had any impact in reducing corruption is still a relatively new area for research and evaluation”. Anti‐corruption practitioners are, indeed, still trying to understand how to best translate principles such as sanctions, control, transparency, and accountability into reforms and programs against corruption (Johnsøn & Søreide, 2013).

Studies on corruption, even though numerous, have remained quite descriptive with a usual focus on definitions or theoretical issues. Much of the literature leads to conclusions sometimes too general and vague to inspire policy‐makers, or based on assumptions that have not been empirically tested.

Several studies address the effects of anti‐corruption programs in a qualitative way. For example, Disch et al. (2009) qualitatively review 150 projects to highlight “what works and what doesn't” in preventing corruption. Among qualitative evaluation projects we can also include different studies of the World Bank and the Organization for Economic Co‐operation and Development (OECD) that have deployed important efforts and resources in evaluating the state‐of the‐art in research and analysis of anti‐corruption and transparency. For instance, the study prepared by the Independent Evaluation Group of the World Bank (IEG Working Paper 2008/7) describes the challenges, effects and limits of the World Bank Support program in promoting anti‐corruption initiatives, drawing on the results of 19 country case studies covering developing and transitional countries (Fjeldstad and Isaksen, 2008). The OECD country reports monitor the implementation of the OECD Anti‐Bribery Convention^1^ and the progress in preventing corruption of countries participating in the Istanbul Anti‐Corruption Action Plan.^2^

Besides these qualitative evaluations, there has been a serious lack of systematic and comprehensive assessments of the implementation and effectiveness of anti‐corruption measures. In this field of research, there exists only the systematic review of Hanna et al. (2011) on “The effectiveness of anti‐corruption policy”. The study has demonstrated that existing research on the effectiveness of anti‐corruption measures present two major shortcomings: (1) They are mainly focused on the incentives and advantages of not engaging in corrupt practices, but there remains a dearth of knowledge in relation to the assessment of public sector reforms adopted to reduce the “need” (or the “market”) for corrupt behavior. (2) They use a very broad definition of corruption, which sometimes even includes theft and fraud.

Drawing on the above mentioned shortcomings, and on Hanna's conclusions, the main objective of this review is to empirically evaluate the effectiveness of anti‐corruption measures in the public sector and to investigate whether and how control and deterrence interventions have different impacts on administrative corruption than organizational and cultural reforms. Furthermore, the review also explores whether the impact of these anti‐corruption policies vary across high‐, middle‐ and low‐income countries and across different public sectors (e.g., education, health, public administration).

In this sense, our study offers a bridge between theory and literature of anti‐corruption strategies on the one hand, and their empirical effectiveness on the other.

### 2.1 The problem, condition or issue

#### 2.1.1 Administrative Corruption

Since the majority of anti‐corruption interventions and reforms target public corruption at the State administrative level (USAID, 2009, World Bank 1997: 4; European Commission, 2014), this review focuses on administrative corruption.

Corruption is a multifaceted and evolving phenomenon that involves different types of actors, behaviors and activities. The concept is broad and there is no consensus on a standard and exhaustive definition. The complexity of corruption cannot be dealt with unidimensional and general definitions but only with a multi‐disciplinary approach (Mugellini, 2020).

Nonetheless, the most widely adopted definition of corruption is the one developed by Transparency International (2013): “the abuse of entrusted power for private gain”. This conceptual definition constitutes a broader version of those provided some years before by the World Bank (1997)—“use of public office for private gain”—and by the Organizations for Economic Co‐operation and Development‐OECD (2008)—“the abuse of a public or private office for personal gain”. The main issue with Transparency International's definition, is an over‐simplification of the phenomenon and the lack of a clear understanding of the specific behaviors constituting corruption.

In order to overcome this issue, several classifications of corruption have been developed in the past decades to ease the identification of corrupt behaviors by grouping them on the basis of similar mechanisms and attributes (Mugellini, 2020).

Among them, the distinction between administrative and political corruption is one of the most common. This distinction is related to the types of operation influenced by the corrupt behavior and to the types of public official involved (Mugellini, 2020).

Administrative/bureaucratic corruption concerns corrupt acts involving civil servants/bureaucrats in their dealings with either their superiors (e.g., bribery to obtain a career advantage, or to hire relatives) during the implementation of public policies, or while interacting with the public (either private citizens or business representatives) for service delivery (e.g., bribery to get a license or avoid a fine) (Gould, 1991; Huberts, 1998: 211; Khan, 2004; OECD, 2015; Pope, 2000; Riccardi & Sarno, 2013, : 19‐ 29; Mugellini, 2020). Political/legislative corruption mainly affects legislators and influences the formulation of laws, regulations, and policies (Kramer, 1997; OECD, 2015; Rose‐Ackerman, 1978: 28; The World Bank, 2003: 6).

In the words of Zhang and Vargas‐Hernández (2017: xiv), “administrative corruption is the most widespread type of corruption and it is sometimes treated as a narrow term of public corruption”.

Administrative corruption can involve public institutions in the strict sense as well as quasi‐private organizations with a strong link to the public sector, either because their mandate is given by the State or because the State is the main shareholder. These public‐private arrangements prompt types of corruption which are closer to those of private sector and can be more easily prevented and punished through internal mechanisms compared to corruption in the public sector. Hence, they are not considered in this review.

Most administrative corruption takes place at the implementation end of public policies, although it may in some cases have its roots in the planning and budgeting stages that precede implementation (Isaksen, 2005). It involves appointed bureaucrats and public administration staff at the central or subnational levels. This includes interaction with private agents, such as demanding extra payment for providing government services, extra‐money to expedite bureaucratic procedures, or bribes to allow private actions that violate rules and regulations. It also includes interaction within the public bureaucracy, such as bribes or kickbacks to obtain jobs or secure promotion, or mutual exchanges of favors. This type of corruption is often referred to as petty corruption, which reflects the small payments often involved, though in specific cases and in aggregate, the sums may be large (Blundo et al. 2006).

Administrative corruption also refers to the intentional imposition of distortions in the prescribed implementation of existing laws, rules and regulations to provide advantages to either state or non‐state actors. Example of administrative corruption are bribes to an official inspector to overlook minor (or possibly major) offences of existing regulations, bribes to gain licenses or to smooth customs procedures (World Bank, 2000).

The “Study on Anti‐Corruption Measures in EU Border Control” describes administrative corruption as follows: “*administrative/bureaucratic corruption is related to manipulation of public tenders, kickbacks from providers, nepotism‐based recruitment and promotions*”. This study also highlights that “*significant funds are being allocated by the EU cohesion policy to the strengthening of administrative capacity at all levels, including regionally, especially in less developed regions and newer EU Member States. The added administrative efficiency that should result will reduce actual levels of corruption and consequently the pressure on personnel to become corrupt. Once administrative efficiency has been improved, additional specific anti‐corruption measures can be added*.” (Centre for the Study of Democracy, 2012: 7‐8).

The United Nations Convention Against Corruption (UNCAC) identifies 11 different types of activity that should be criminalized as corruption by State parties in their jurisdictions (United Nations Office on Drugs and Crime‐UNODC, 2004: 17–19). Six of them can be classified as types of administrative corruption: bribery of national public officials; bribery of foreign public officials and officials of public international organizations; misappropriation or other diversion of property by a public official (embezzlement), trading in influence, abuse of function, illicit enrichment (UNODC, 2004: 17‐19).

Other behaviors that are not criminalized but which could possibly lead to corruption must also be considered for a proper and comprehensive classification of administrative corruption (Villeneuve et al., 2019). Favoritism, for example, understood as the human inclination to prefer acquaintances, friends and family over strangers, is not a type of corruption *per sè* (Esadze, 2013), but it leads to corruption when it is used by officials to unfairly distribute positions and resources without regard to merit. Three main types of corruption can arise from favoritism depending on the relationship between the official and the person who benefits from his/her favor. Cases where family members are favored are considered as cases of nepotism. Favoring close friends is defined as cronyism (Villeneuve et al., 2019). When political parties or supporters are rewarded for their support through a job or a government benefit, patronage occurs (Esadze, 2013; Graycar, 2013).

Our proposed research refers to the administrative corruption in the public sector as the abuse of public office or public role by for private gain by civil servants/bureaucrats in their dealings with either their superiors (e.g., bribery to obtain a career advantage, or to hire relatives), during the implementation of public policies, or while interacting with the public (either private citizens or business representatives) for service delivery (e.g., bribery to get a license or avoid a fine). By civil servants/bureaucrats we refer to people belonging to any kind of public institutions (e.g. schools, hospitals, etc.) in addition to governmental ones, with the exclusion of politicians.

In particular, this research considers the following main corrupt acts: bribery of public officials; misappropriation or other diversion of property by a public official (embezzlement), nepotism, cronyism, trading in influence, abuse of function, illicit enrichment. Some corruption‐related concepts were also included in the review such as fraud. Fraud is a broader legal and popular term that covers both bribery and embezzlement. Furthermore, the notion of absenteeism as a serious form of corruption in specific sectors, such as education and health (Transparency International 2013) is also considered for this research. Indeed, “stealing time” (e.g. not show up at work) while performing a public office is considered a serious form of diversion or theft of state assets (time in this case) (Hanna 2011: 8).

Although administrative corruption is often defined and studied together and as opposed to political corruption, this proposed study excludes political corruption. We deliberately want to separate politics from administration. Political corruption affects the formulation of policies; it involves political decision‐makers and takes place when politicians, who are entitled to make the laws (the rulers), are themselves corrupt. In political corruption, laws and regulations are abused and even tailored to fit the interest of the rulers. This type of corruption also includes “vote‐buying” (Rose‐Ackerman, 1978). Political corruption takes place at the high levels of the political system and it has political repercussions (Amundsen, 1999). Administrative corruption occurs at any level of authority in the public administration and affects the implementation of policies. The study does not examine the acts of political corruption mainly because we believe that the mechanisms and the functioning of political corruption (i.e. manipulations of rules of voting systems) are significantly different and more complex than those related to administrative corruption. We thus believe that political corruption would need an ad‐hoc separate review and evaluation.

This study differentiates from the one of Hanna et al. (2011) by (1) including only experimental studies (RCT) (2) considering both developing and developed countries (classified in low, middle and high income countries according to the World Bank classification), (3) including a wider range of interventions, (4) addressing both single anti‐corruption policy and combinations of anti‐corruption strategies and (5) by using meta‐analysis instead of textual narrative review.

### 2.2 The intervention

#### 2.2.1 Anti‐Corruption Policies in the public sector

Reforms are changes or adjustments that are well‐studied and planned with a clear objective of improving the current state of the elements part of a system Caiden (1969).

Anti‐corruption policies developed in the public sector are reforms aimed to prevent or eradicate corrupt behaviors in public administration and service delivery. The main objective of anti‐corruption policies is to increase the effectiveness, transparency and accountability of the public sector through improved administrative, financial and control systems. This characteristic makes anti‐corruption efforts implicitly embedded into broader governance reforms, and a sort of “by‐product” of public sector reform (Chên, 2008; Hussman, 2007).

McCusker (2006) advocates that there are three possible arenas for anti‐corruption reforms: (1) agenda setting – many governments have yet to recognize corruption as a serious problem, far fewer governments place it on their national agenda; (2) decision‐making – attempts to get anti‐corruption reforms approved yet alone implemented have been mixed; (3) implementation – many reforms that have succeeded in being enacted have encountered obstacles in execution, often preventing the effective resolution of the problem corruption (Tay & Seda, 2003).

The OECD (2003, p. 4) indicates four main types of anti‐corruption policies. 1) Prevention in a repression perspective aims at increasing the transparency of public operations, through for instance the adoption of measures to facilitate access to information. 2) Prevention in an incitation perspective aims at changing the logics of action that lead public or private actors to bribery. For example, managing conflicts of interest in public service allows protecting the integrity of official decision‐making. 3) Detection aims at defining and supporting the role different actors can play in detecting potential cases of corruption (e.g., whistleblowing mechanisms, tax inspectors, auditors). 4) Repression aims at defining offenses, directly or indirectly linked to corruption and setting up State mechanisms to investigate and sanction them.

Huberts (1998) distinguishes six areas of anti‐corruption strategies: (1) economic – a strategy which suggests paying higher salaries to civil servants; (2) educational – through training and education campaign, this strategy aims at changing the attitude and values of civil servants; (3) cultural – a strategy which ensures ethical codes of conduct for civil servants; (4) organization and bureaucratic – enhancing internal control systems to detect corrupt activities; (5) political – increasing in the transparency of the monitoring of party finances and strengthening the separation of powers in terms of the judiciary and the state; (6) judicial or repressive measures – aims at harsher penalties for corrupt practices but also the creation of independent anti‐corruption agencies. Huberts’ (1998) classification is focused on the specific content and characteristics of different anti‐corruption reforms and it is based on the views of 257 experts from 49 countries with very different political, economic and societal conditions.

Several other classifications of anti‐corruption policies have been developed in the past decades (see for example, Blind, 2011; Brunetti & Weder, 2003; Dish et al., 2009; Graycar, 2015; Holmes, 2015; Lambsdorff, 2009; Lange, 2008; McCusker, 2006).^3^

Taking OECD's (2003) and Huberts’ (1998) classifications as starting point and considering the characteristics of the other existing classifications, we identify two main categories of anti‐corruption interventions: 1) Control and deterrence interventions based on increased punishment (e.g. higher sanctions for corrupt officials), increased control (e.g. auditing systems) and positive incentives (e.g. premiums form competent and rapid service to citizens), and 2) Cultural and organizational interventions based on cultural and ethical education of public officials (e.g., codes of ethics, regular trainings, sensitization messages, etc.), and organizational changes (e.g. decentralization, regular staff rotation).

The choice of focusing on these two categories can be justified from an empirical and theoretical point of view. Indeed, these two domains are able to capture the majority of anti‐corruption interventions considered in the literature, and, at the same time, allow for a parsimonious statistical model.^4^

Furthermore, these two categories distinguish interventions according to two main theoretical explanations of corruption: the economic and the cultural paradigm.

Control and deterrence interventions mainly draw on economic explanations of corruption rooted in the principal‐agent model (Rose‐Ackerman, 1978). According to this model, corruption can be interpreted as the outcome of cost‐benefit reasoning, of a rational choice. In this context, deterrence is considered a way to increase the risks or costs of misbehaviors in rational and economic calculations (Rose‐Ackerman, 1978; Zimring & Hawkins, 1973, and Klitgaard, 1988). Rewarding compliance with specific rules by providing positive incentives also belongs to this logic. Therefore, control and deterrence anti‐corruption interventions aim to: a) increase the probability of discovering the corrupt behavior by increasing monitoring; b) increase the punishment for people engaging in corrupt activities; c) increase incentives to promote compliant behavior. A few studies have demonstrated that monitoring and deterrence programs had a significant effect on corruption only when they are simultaneously developed (Hanna 2011). For example, the study of Di Tella and Schargrodosky (2003) demonstrated that the effect of economic incentives for public officials on corruption is negative and significant only when associated to audits systems (Di Tella and Schargrodosky 2003).

Cultural and organizational interventions rely on the cultural and neo‐institutional theoretical paradigm for explaining corruption (Vannucci, 2015). These paradigms do not consider the background or motives of the corrupt individuals but rather the environmental characteristics, structure and culture of the organizations where public and private agents work or live, and the related group behaviors that might foster corruption (De Graaf, 2007, p. 52), together with the institutional framework regulating social interactions. This type of interventions starts from the assumption that monitoring or punishments are easily bypassed (Banerjee et al., 2007), while a structural change in the organization, culture, and rules of specific services can reduce the “market”, “need” and “justification” for corruption (Marquette & Pfeiffer, 2015). Therefore, organizational and cultural interventions aim to: a) change the attitude and values of civil servants by training and education campaign; b) increase the culture of legality by developing ethical codes of conduct for civil servants; c) increase transparency and dissemination of information by public administration; d) improve the efficiency of public services, e) avoid conflicts between an individual's private interests and his/her public role.

Detailed examples of how these interventions work are provided in the following section “How the intervention might work”.

### 2.3 How the intervention might work

The universe of anti‐corruption interventions and reforms is vast and, depending on the type of strategy and approach, on the type of actors involved, and the environment where they have been developed, these interventions might work in different ways.

The mechanisms exploited by the two categories of interventions originate from the different theoretical understandings of corruption.

Control and deterrence interventions, based on economic theories that interpret corruption as the outcome of cost‐benefit reasoning, work by increasing the risks or costs of engaging in corrupt behaviors. This group of interventions can function in the following ways: a) by increasing the probability of discovering the corrupt behavior through increased monitoring; b) by increasing the punishment for people engaging in corrupt activities; c) by increasing incentives to promote compliant behavior.

Cultural and organizational interventions, based on a cultural and neo‐institutional understanding of corruption that consider corruption more a result of the environmental characteristics, structure and culture of the organizations where public and private agents work or live, and the related group behaviors, mainly work by: a) changing the attitude and values of civil servants by training and education campaign; b) increasing the culture of legality by developing ethical codes of conduct for civil servants; c) increasing transparency and dissemination of information by public administration; d) improving the efficiency of public services, e) avoid conflicts between an individual's private interests and his/her public role.

**Figure 1:**
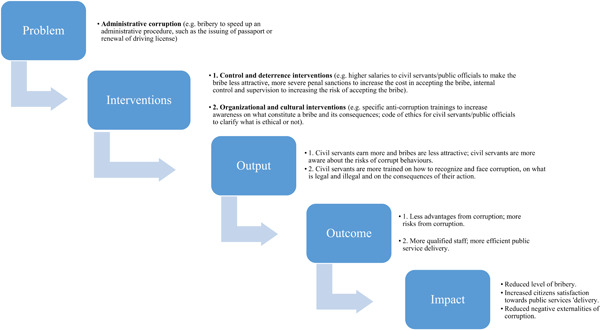
Example of the practical functioning of anti‐corruption interventions in public administration

Figure 1 below provides an example of the practical functioning of control and deterrence, and organizational and cultural interventions.

**Figure 2:**
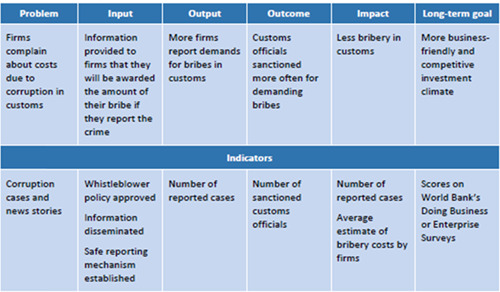
Example of the practical functioning of anti‐corruption interventions. Source: Johnsøn and Søreide (2013: 6)

A more detailed and illustrative example of how a specific types of control and deterrence anti‐corruption reforms might work is provided by Johnsøn and Søreide (2013: 6) (see Figure 2 below).

The authors focus on the process of a program aimed at reducing corruption in customs by offering a reward to businesses who report they have paid a bribe. For each step of the process, specific measurable indicators are also provided to show how the impact of this intervention might be empirically measured. However, Johnsøn and Søreide (2013) also highlight that it is not always easy to identify and evaluate the working process of a specific intervention, as it might be difficult to recognize preconditions and intervening variables. The idea is, therefore, to go beyond the log‐frame approach and to consider also the socioeconomic and political context of the intervention to understand how it worked, and thus properly assess its effects. This is how our study is going to proceed.

A practical examples of organizational anti‐corruption reform suggested by Schleifer and Vishny (1993; 610) is to produce competition between bureaucrats in the provision of government goods. This arrangement has been introduced in many agencies of the US government, as passport offices. It seems that creating competition in the provision of government goods might increase theft from the government but at the same time reduces bribes.

Decentralization and the use of electronic payments are other examples of organizational anti‐corruption reforms. When applying a decentralization policy, the responsibility for the implementation of a given policy passes from a higher level of government to a lower one (Hanna 2011: 9). The use of technology and electronic payments can help to bypass various lengthy bureaucratic procedures and to avoid direct contact with civil servants. This can reduce the opportunity for bribes (Hanna 2011).

In the case of Italy, the new anticorruption law ratified in November 2012 was specifically addressed to the reduction of administrative corruption through the increase of transparency and dissemination of information by the public administration. Within the three‐year national anti‐corruption and integrity action plan addressed to all administration bodies, both control and deterrence interventions (i.e., increasing the detection of corruption cases by reinforcing whistleblowers’ protection through the development of informatics systems within public administration to reporting any suspected operation online (European Commission, 2014, p. 4)), and organizational and cultural interventions (i.e., Public e‐Procurement system to manage public bid processes online and increase transparency (Acquistiinretepa.it); regular shifts of management staff; development of code of ethics for the public administration (European Commission, 2014, p. 9)) have been developed.

The types of interventions vary also on the basis of the country where they are developed. For example, Public Expenditure Tracking Surveys (PETS) are considered among the few methods having a positive impact on corruption in service delivery in developing countries with a weak system of governance (Sundet, 2008, p. 2). The application of PETS in Uganda, for example, shows that the flow of funds improved dramatically, from 13 percent on average reaching schools in 1991‐95 to around 80 percent in early 2001 (Reinikka & Svensson, 2004). However, this method was not successful in other countries with similar characteristics (e.g. in Tanzania).

### 2.4 Why it is important to do the review

Considering that “the absence of rules facilitates the process of corruption as much as the presence of cumbersome or excessive rules does” (Bank's General Counsel, Ibrahim Shihata), an evaluation of what works and what does not in curbing corruption is of extreme importance in order to identify the most effective intervention or combination of interventions.

Despite a large amount of literature on anti‐corruption, there are few systematic reviews focusing on anti‐corruption reforms and even fewer assessing the issues of effectiveness and impact. There is thus a need for more comprehensive assessments of the implementation and the efficiency of anti‐corruption measures in different settings.

Therefore, the main objective of this review is to systematically identify any evidence and evaluation of the effectiveness of different anti‐corruption reforms in the public sector. Anti‐corruption policies or interventions cannot operate successfully without a concrete assessment of their impact on the level of corruption. It is necessary to know exactly what has worked, what has been useful so far, what has not worked and how to do better in the fight against corruption.

Our systematic review identifies studies presenting anti‐corruption reforms and interventions and comprehensively assessing their outcomes.

The proposed research has a main impact for the criminological and criminal law field but, as far as corruption issues and anti‐corruption policies concern many other disciplines, it also contributes to advance research in political sciences, economics, public administration, etc. In addition, our review clarifies in what field of research empirical studies are concentrated (for instance what kinds of interventions and outcomes). Finally, given the lack of assessments of this sort at the international level, such review will be of high relevance to the international literature on administrative anti‐corruption strategies.

One major contribution to the subject is the systematic review of Hanna & al. (2011) on “The effectiveness of anti‐corruption policy”. This review demonstrates that the major shortcoming of existing research on the effectiveness of anti‐corruption measures is related to the limited focus on the incentives and advantages of not engaging in corrupt practices, but there remains a dearth of knowledge in relation to the assessment of public sector reforms adopted to reduce the “need” (or the “market”) for corrupt behavior. The review of Hanna focuses on developing countries, on two main types of interventions (i.e. monitoring and incentives mechanism, and changing the rules of the system) and it uses a textual narrative synthesis approach. Hanna stated that there would have been the need to better understand the long‐term effects of anti‐corruption strategies focused on the changes of the rules and on those more oriented to monitoring and incentives interventions (Hanna 2011: 1).

Our review will differentiate from the Hanna's one by (1) including only experimental studies (RCT) (2) considering both developing and developed countries (classified in low, middle and high income countries according to the World Bank classification), (3) including a wider range of interventions, (3) addressing both single anti‐corruption policy and combinations of anti‐corruption strategies and (4) by using meta‐analysis instead of textual narrative review. As far as the features and mechanism for corruption change quickly, as well as the measures for countering this issue, this systematic review will also serve as an update to the results of the previous studies.

When it comes to study causal relationships, experimental studies are known to be the more valid ones. Randomization allows to control for any potential confounder and so experimental studies allow us to make causal inference, whereas observational studies cannot. Furthermore, including other types of empirical studies would have likely increased the between‐study heterogeneity, making conclusions somehow less reliable, as studies included in a meta‐analysis should share similar underlying characteristics. Furthermore, if more qualitative studies are included, it would have been impossible to perform a meta‐analysis.

Considering both developing and developed countries is fundamental to identify potential variations in the effectiveness of anti‐corruption interventions related to different contextual and socio‐economic characteristics. Graycar and Monaghan (2015) highlight that anti‐corruption policies are mostly centered on features of corruption found predominantly in developing countries and leave behind other relevant determinants of corruption in developed countries (Graycar & Monaghan, 2015). The inclusion of both also allows to overcome this recent critic to the study of anti‐corruption efforts.

Textual narrative reviews can give important insights into the mechanisms behind a certain causal relationship, but meta‐analysis also allows researchers to quantify the effect of an intervention. This is very important from a policy point of view, when the need is to identify effective interventions that bring about substantial improvements. Although widely adopted, narrative reviews entail several issues. Authors might be biased due to their subjectivity (Green et al., 2006; Stanley & Jarrell, 1989). By choosing which studies to include in the review, how to weight them and how to interpret their results, reviewers can accommodate, even unconsciously, the main findings of a particular scientific field to be com‐pliant with their prior beliefs (Stanley & Jarrell, 1989).

Moreover, exploiting the bigger sample size obtained by merging all studies considered in this review, meta‐analysis allows us to get more precise estimates of the effect of interest.

## 3 OBJECTIVES

The main goal of this review is to empirically evaluate the impact of anti‐corruption measures, developed in the public sector, on administrative corruption.

In order to pursue this goal, this project synthetizes and compares the results of published and unpublished studies providing empirical evidence on the effects of different anti‐corruption interventions, developed in the public sector, to counter administrative corruption.

The study considers two main categories of interventions – control and deterrence, and organizational and cultural ‐ that are able to cover the majority of existing anti‐corruption measures, to adhere to the main theoretical frameworks on corruption and, at the same time, to guarantee the reliability of the meta‐regression model.

While pursuing the above‐mentioned main goal, this project also considers the following objectives:

1. To identify which macro‐category of interventions (control and deterrence; organizational and cultural) have a significant effect on the level of administrative corruption.
2. To examine whether specific interventions (e.g., economic, educational, organizational, legal, etc.) have different effects on the level of administrative corruption.
3. To assess whether and how the effects of the interventions vary across high‐ and low‐income countries.
4. To determine whether and how the effects of the interventions vary by type of corruption (e.g. bribery vs misappropriation of public resources; nepotism vs extortion).
5. To check whether and how the effects of the interventions vary by type of public sector (e.g. public procurement, education, health, construction).

## 4 METHODS

In order to fulfill the above mentioned objectives, we reviewed existing published and unpublished studies covering different interventions against administrative corruption. We collected and synthetized evidence on the effectiveness of these interventions by using transparent procedures to locate, evaluate, and integrate the findings of the relevant research, and rigorous statistical analysis. The methodological process have been developed following the standards and principles of systematic reviews, in order to ensure accurateness, methodologically soundness, comprehensiveness, and control for risk of bias.

### 4.1 Criteria for considering studies for this review

#### 4.1.1 Types of studies

Studies eligible for inclusion in this review quantitatively assess the effects of interventions in the public sector on the level of administrative corruption. Only studies based on randomized control trials (RCTs) are included in this review. We do not discriminate between field and laboratory experiments. Armantier and Boly (2008) tested the external validity of corruption experiments by moving from the lab in a developed country to the field in a developing country and found out that laboratory experiments, in the anti‐corruption research field, seem to have a quite high degree of external validity. In particular, they run the same experiment in the laboratory in Canada and in the field in Burkina Faso and found out that, after controlling for individual characteristics, the direction and magnitude of most treatment effects were statistically indistinguishable between the lab and the field.^5^ In a subsequent study on the external validity of laboratory experiments on corruption, the same authors (Armantier & Boly, 2011), concluded that “Although a definitive answer to the external validity question has yet to be provided, these preliminary results provide some support to the external validity of lab experiments on corruption.”

Whang (2009: 24) explains the reasons why laboratory experiments allow to control the behavior of subjects in a way that is not possible in the field. First of all: “When modelling a strategic real‐life environment, a theorist relies on behavioral assumptions, typically the assumption of fully rational profit maximization. If these assumptions are not met, the theoretical results may be distorted. Experimental methods are used to test theoretical models. In a laboratory, a rigorous test of the behavioral underpinnings of the model can be carried out” (Whang 2009: 24). Secondly: “laboratory experiments allow researchers to address the issue of causality in ways not possible in field contexts. Thus in studying corruption, the laboratory is an easily controlled environment where it is possible to isolate the specific features that can be at play when subjects send and accept bribes. Therefore, we can design experiments that mimic specific aspects of corruption scenarios, although in a simplified version, to address the issue of causality.” (Whang 2009: 25). The author concludes by considering that although laboratory experiments cannot replicate exactly the complexities of the real‐world policy making, their results are still informative (Whang 2009: 25).

Furthermore, Camerer (2012: 8) states that: “parallelism does not require that students in a lab setting designed to resemble foreign exchange behave in the same way as professional foreign exchange traders on trading floors…. The maintained assumption of parallelism simply asserts that if those differences could be held constant (or controlled for econometrically), behavior in the lab and the trading floor would be the same”.

The results of our meta‐regression analysis confirms there are no differences between RCTs conducted with students as participants and those with other types of participants.

Natural experiments are instead excluded to increase the methodological homogeneity of the sample of studies. In fact, unlike RCTs and quasi‐experimental studies, within the natural experiment framework researchers cannot randomly assign participants to control and treatment groups; they are selected based on the exposition to “natural” factors (e.g. policies). This might raise warnings about unaccounted confounding effects. Following well‐known guidelines to perform systematic reviews, strict methodological criteria have been established. Studies for being included had to:

- Include at least two distinct groups: a group exposed to the anti‐corruption intervention/s and a control group not experiencing the intervention/s;
- Contain at least one outcome measure of corruption (e.g., bribe demand, bribe offer, embezzlement, favoritism);
- Consider anti‐corruption intervention/s developed within the public sector (e.g., public administration, education, health, construction).

We only included studies written in or translated into English because we noticed that experimental studies in this field of research are usually translated into English to ensure their results’ dissemination.

No other restriction in terms of type of publication, subject area, field, and geographical area, type of public sector, age, and gender of participants has been applied. All studies published until 2018 have been considered for inclusion.

#### 4.1.2 Types of participants

As far as anti‐corruption policies can address both the suppliers and consumers of public services, the studies included in this review involve both civil servants/public officials and citizens, as participants.

By public servants, we refer to people belonging to any kind of public institutions (e.g. schools, hospitals) in addition to governmental ones, with the exclusion of politicians.

In case of laboratory experiments, no restriction has been applied with regard to type of persons acting in the roles of public officials or citizens. The majority of the included lab experiments involve students as participants, with few exceptions that conduct lab experiment with public servants. The types of participants to the lab experiment have been included as covariate in the meta‐regression analysis and showed no robust significant impact on the outcome variable.

No restriction about geographical area has been applied. Studies have been grouped into four categories depending on the level of national income of the countries where the experiment was developed. The World Bank classification of the level of national income has been taken as a basis. For the current 2019 fiscal year, low‐income economies are defined as those with a GNI per capita of $995 or less in 2017; lower middle‐income economies are those with a GNI per capita between $996 and $3,895; upper middle‐income economies are those with a GNI per capita between $3,896 and $12,055; high‐income economies are those with a GNI per capita of $12,056 or more.^6^

#### 4.1.3 Types of interventions

We consider studies including an evaluation of anti‐corruption policies developed in the public sector and targeted at administrative corruption. The review includes studies focused on interventions that have had direct effects on the level of administrative corruption (as defined above), without any geographic and temporal limitations. The anti‐corruption policies considered for this review have been grouped into two main categories: 1) Control and deterrence interventions based on increased punishment (e.g. higher sanctions for corrupt officials), increased control (e.g. through auditing systems) and positive incentives (e.g. premiums form competent and rapid service to citizens), and 2) Organizational and cultural reforms based on organizational changes (e.g. decentralization, regular staff rotation) and the ethical and cultural education of public officials (e.g., codes of ethics, regular training, sensitization messages, etc.)

To be included in the review studies had to measure quantitatively the anti‐corruption intervention either as a dichotomous variable (e.g. presence/absence of a specific reforms), ordinal variable (e.g. low, medium and high level of monitoring), or as a cardinal variable (e.g. increased wages for public officials).

The classification of anti‐corruption interventions was also expanded to six sub‐categories (as defined by Huberts, 1998): (1) economic; (2) educational; (3) cultural; (4) organizational; (5) political; (6) judicial or repressive measures. However, no robust results were found for this level of disaggregation.

#### 4.1.4 Types of outcome measures

##### Primary outcomes

The dependent variable is the level of administrative corruption. Administrative corruption is intended as those corrupt acts involving civil servants/bureaucrats in their dealings with either their superiors, during the implementation of public policies, or while interacting with the public for service delivery. According to the United Nations Convention Against Corruption (UNCAC 2004), it includes acts of bribery, extortion, misappropriation or other diversion of property by a public official (embezzlement), theft of state assets or diversion of state revenues, absenteeism, favoritism.

The included studies address four main types of administrative corruption: active bribery, passive bribery, embezzlement and favoritism. Following UNCAC definitions, the former refers to the “promise, offering or giving, to a public official, directly or indirectly, of an undue advantage, for the official himself or herself or another person or entity, in order that the official act or refrain from acting in the exercise of his or her official duties” (UNCAC, 2004, Art. 15, (a)). The latter refers to the “solicitation or acceptance by a public official, directly or indirectly, of an undue advantage, for the official himself or herself or another person or entity, in order that the official act or refrain from acting in the exercise of his or her official duties” (UNCAC, 2004, Art. 15, (b)). The distinction between active and passive bribery aimed to catch any potential difference among the impacts of anti‐corruption policies depending on the passive or active role of the public servants.

Embezzlement is considered as the misappropriation, theft or other diversion, by a public official, of property, public or private funds or securities or any other thing of value entrusted to the public official by virtue of his or her position (UNCAC 2004, Art. 17).

Favouritism is understood as situations when the public official prefer acquaintances, friends and family over strangers to unfairly distribute positions and resources without regard to merit (Esadze, 2013).

The four types of corruption have also been grouped into two macro categories: “extortionary” and “collusive” corruption. Extortionary or coercive corruption concerns situations when citizens have to pay bribes for services they are entitled to receive (e.g., getting a driver's license, a birth certificate, registering a purchase of property; the transaction does not generate negative externalities to others) (Ryvkin et al., 2017). Collusive corruption happens when a bribe is exchanged for the provision of an illegal good or service, for instance for the provision of a building permit to an unqualified firm. It may impose negative externalities on the society (poorly constructed bridge that passes inspections and then breaks down) (Ryvkin et al., 2017). This distinction has been introduced to understand whether the provision of legal or illegal services has different mechanisms and is affected by different types of policies or not.

The assessment of the level of corruption before and after the intervention referred only to experiences of corruption and not to its perception.

Due to the multifaceted nature of corruption, the identified categories of corruption are not perfectly mutual exclusive and may overlap over specific characteristics. However, one “dominant” characteristic was always identified and attributed to only one macro category of corruption.

##### Secondary outcomes

We are aware that, besides more direct impacts such as the reduction in the level of specific forms of corruption, interventions might also have indirect effects such as improved public integrity, improved quality or quantity of specific public services (Banerjee et al., 2010; Björkman & Svensson, 2009). Usually, the latter is a consequence of the former and they are identifiable only in the long‐term period. It can also happen that interventions originally addressed to the improvement of a specific public service, had an impact on the level of corruption. Also, we understand that the outcome of specific anti‐corruption reforms might have a wider positive displacement than simply reduce the level of corruption. However, the purpose of our review is to focus on interventions with direct effects on the level of administrative corruption. Such a decision helps us not to get lost in other types of outcome indicators, such as public service quality or access to public service. Therefore, the type of outcome or the dependent variable of this review is the level of administrative corruption.

#### 4.1.5 Duration of follow‐up

No restriction on the duration of the follow‐up have been defined.

#### 4.1.6 Types of settings

No restriction on the types of settings have been defined.

### 4.2 Search methods for identification of studies

#### 4.2.1 Electronic searches

The literature search was conducted using three highly recognized electronic databases: RePEc, SSRN, Web of Science. These databases are considered the most comprehensive in the socio‐economic field of research.

In addition, the following grey literature databases have been searched:

- Campbell Crime and Justice Group
- IDEAS (Internet Documents in Economics Access Service)
- NBER (National Bureau of Economic Research)
- Networked Digital Library of Theses
- Dissertation Index to Theses,
- DocTA (Doctoral Theses Archive)
- Proquest's Digital Dissertation
- Rutgers Grey Literature Database

Both published and unpublished studies were searched based on the keywords combinations indicated in the online supplement 1.

#### 4.2.2 Searching other resources

A reverse snowballing approach on Google was also performed in order to screen the references of the identified studies. Titles of eligible papers were also searched in Google Scholar in order to identify potential articles quoting them. In order to ensure replicability, all searches were stored into Covidence, an online software product developed by Cochrane community for screening studies and extracting data for systematic reviews.

### 4.3 Data collection and analysis

#### 4.3.1 Selection of studies

Two independent review authors read the titles and abstracts of identified studies in order to determine their eligibility against the inclusion/exclusion criteria. When a title or abstract could not be rejected with certainty, the full text of the article was reviewed. In case of disagreement about whether or not a study should have been included, the lead author, Dr. Giulia Mugellini, together with Martin Killias, acted as arbitrator. The included studies were then organized into categories based on the study design, type of interventions, type of administrative corruption, level of national income. Studies that did not meet the inclusion criteria are listed in the References of excluded studies. The reasons for exclusion are reported in Table 3.

### 4.4 Data extraction and management

The relevant information of identified studies was extracted independently by two review authors, following the guidelines of the Campbell Collaboration.

In particular, the coding sheet contains information related to the references of the study, experiment characteristics, outcome measure (proxy of corruption), effect seizes, econometrics, types of interventions, type of sector, study quality (see Table 1 below for the complete coding sheet).

**Table 1:** Coding Sheet

**References of the study**:
study: study numeric ID; numeric characters.
case: case per study; alphanumeric characters (1‐A, 1‐B, 2‐A, 3‐A, 3‐B, 3‐C…)
author: short author‐year ID. If 1 author code as “surname1 (xxxx)”, 2 authors as “surname1 & surname2 (xxxx)”, 3 or more author as “surname1 et al. (xxxx)
apa: APA full reference
journal: journal in which the study has been published short capital form; if it is a book or it is not available it will be coded as NA
year: year included in the APA reference; please refer, every time possible, to the year of the publication if more than one version (e.g. previous working paper) is available
**Experiment characteristics:**
country: country in which the treatment has been conducted
low.income: dummy = 1 if the study is conducted in a low income country, 0 otherwise. Classification is from https://data.worldbank.org/products/wdi-maps
low.middle.income: dummy = 1 if the study is conducted in a low‐middle income country, 0 otherwise.
upper.middle.income: dummy = 1 if the study is conducted in a upper‐middle income country, 0 otherwise
high.income: dummy = 1 if the study is conducted in a high income country, 0 otherwise
subjects: number of subjects involved in the experiment
sessions: number of experimental sessions
laboratory: = 1 if the study is conducted in a lab, 0 otherwise (field‐experiment)
student: = 1 if all the participants in experiment are students, 0 otherwise
**Xi:**
Contains the regressors (beta coefficient), SE and t‐stat of the experiment. We code each specification from the top to the bottom in case of multiple variables/treatment in the same specification. Degrees of freedom are equal to observations – regressors (−1 if the constant is not included among the regressors)
Caution: code the t‐stat and not the absolute t‐stat – i.e. the t‐statistic must have the same sign as the beta coefficient.
**Econometrics:**
observations: number of observation
regressors: number of regressors (includes constant and f.e.)
degree.freedom: degrees of freedom, given by observations – degree.free
endogeneity; dummy =1 if the study accounts for potential endogeneity issue
clustered.se: = 1 if the study clusters the SEs by session or participants, 0 otherwise
conditional: = 1 if the recorded regressor is conditional on another treatment. Describe it in the comment log file.
interaction: = 1 if the recorded regressor is part of an interaction term in the specification stored
estimation: name of the estimator adopted
**Intervention:**
control/deterrence: = 1 if the intervention is based on increased punishment (e.g. higher sanctions for corrupt officials), increased control (e.g. through auditing systems) and positive incentives (e.g. premiums form competent and rapid service to citizens).
organizational/cultural: = 1 if the intervention is based on organizational changes (e.g. decentralization, regular staff rotation; competition between public officials in the provisions of government goods, electronic payments) and/or on the ethical and cultural education of public officials (e.g., codes of ethics, regular trainings, sensitization messages, etc.).
**Type of intervention:**
economic: = 1 if it is focused on the reduction of financial and economic stimuli for corruption (e.g. paying higher salaries to civil servants to reduce vulnerability or temptation to bribes);
educational: = 1 if it is focused on the change of attitudes and values of the population and civil servants (e.g. through training and educational campaigns; increasing public exposure; changing family attitudes population; influencing attitude of public servants; etc.);
cultural: = 1 if focused on the improvement of the ethical standards and examples given by management and on the development of ethical code of conduct for civil servants, as well as on the enhancement of protection for whistle blowers;
organisational: = 1 if it is focused on the improvement of internal control systems and supervision (e.g. auditing systems), decentralization, selection of personnel and rotation of personnel as well as technological improvements helping the organizational system
political: = 1 if it is focused on the improvement of the example given by politicians (e.g. more commitment by politicians to combat corruption.
legal: = if it is focused on the implementation of harsher penalties for corrupt practices but also on the creation of independent anti‐corruption agencies.
**Proxy of Corruption:**
All the variables that are considered important in the relationship banking literature
Bribe.demand.offer: = 1 if the corruption measure is the demand or the offer of a bribe
Bride.paid.accept: =1 if the corruption measures is the payment or the acceptance of a bribe
Embezzlement: =1 if the corruption measures is embezzlement/misappropriation/collusion
Favoritism: = 1 if the corruption measures captures favoritism/nepotism
**Type of Corruption:**
Collusive: =1 if both actors have potential gain from the corruptive actions, 0 otherwise. The bribe is exchanged for the provision of an illegal good or service, for instance for the provision of a building permit to an unqualified firm. It may impose negative externalities on the society.
Extortionary: = 1 if only one actor has potential gains from the corruptive actions, 0 otherwise. Citizens must pay bribes for services they are entitled to receive – getting a driver's license, a birth certificate, registering a purchase of property; the transaction does not generate negative externalities to others.
**Study Quality:**
External.validity.declared: =1 if the study has HIGH external validity as declared by the authors – low risk of bias
External.validity.declared: =1 if the study has HIGH external validity as judged by the coders
Internal.validity: = 1 if the study has HIGH internal validity
Good.quality: =1 if study respect some minimum quality requirements such as: experiment description, number of participants, beta coefficient and measure of dispersion
**Sector:**
public.admin: = 1 if the field in which the intervention is conducted is the PA, 0 otherwise
health: = 1 if the field in which the intervention is conducted is the health sector, 0 otherwise
education: = 1 if the field in which the intervention is conducted is the education sector, 0 otherwise
public.construction: = 1 if the field in which the intervention is conducted is the public construction sector, 0 otherwise

#### 4.4.1 Assessment of risk of bias in included studies

The studies were assessed using the *Cochrane Risk of Bias* checklist as a basis and integrated with additional elements. The final checklist includes the following:

- Sequence generation: Describe the method used to generate the allocation sequence in sufficient detail to allow an assessment of whether it should produce comparable groups.
- Allocation concealment:
- Blinding of participants and personnel
- Blinding of outcome assessors: Describe all measures used, if any to blind outcome assessors from knowledge of which intervention a participant received. Provide any information relating to whether the intended blinding was effective.
- Incomplete outcome data: Describe the completeness of outcome data for each main outcome, including attrition and exclusions from the analysis. State whether attrition and exclusions were reported, the numbers in each intervention group (compared with total randomized participants), reasons for attrition/exclusions where reported, and any re‐inclusions in analyses performed by the review authors.
- Selective outcome reporting: State how the possibility of selective outcome reporting was examined by the review authors and what was found.
- Other sources of bias: State any important concerns about bias not addressed in the other domains in the tool. If particular questions/entries were re‐specified in the review's protocol, responses should be provided for each question/entry.
- Outcome measure (statistical validity)
- Statistical control
- Internal validity
- External validity (representativeness)

During the analysis, it emerged that the most important elements to be taken into consideration for evaluating the risk of bias were the internal and external validity. Indeed, it emerged that not all the above mentioned criteria were observable and/or applicable to the selected studies. Those criteria have been created to address studies in the medical sector, while our review includes social‐economic experiments. Furthermore, as far as this review includes only randomized controlled trials or laboratory experiments presenting some sort of randomization, the internal validity of eligible studies is supposed to be high, and many elements of the Cochrane's checklist became, thus, not relevant. Issues of external validity of our selected studies are mainly related to the representativeness of the samples (see section on the Risk of bias for further details).

#### 4.4.2 Measures of treatment effect

The type of effect size selected for the analysis is the partial correlation coefficient.^7^ It estimates the degree of association between the dependent variable and the independent variable when the other variables included in the model are held constant. It has the benefit of allowing the comparison and the synthesis of the collected estimates when different independent variables having different scales and definitions are used. This situation fits the topics of this systematic review, considering the wide range of anti‐corruption interventions covered by the selected studies. The partial correlation coefficient has been computed as following:

(1) $$r_{f}=\frac{t_{f}}{\sqrt{t_{f}^{2}+d_{f}}}$$

where *t*_*f*_ is the *t* statistic of the regression coefficient *β*_*f*_ while d*f* is the degrees of freedom (*n* − *p* − 1) in which *p* is the number of regressors and *n* is the number of observations. The sample variance of the partial correlation is instead given by:

(2) $$\mathrm{var}\left((,r_{f},)\right)=\frac{{\left((,1-r_{f}^{2},)\right)}^{2}}{n-p-1}$$

the values obtained are then normalized using to the *Fisher's z‐score* transformation, which is equal to:

(3) $$z=\frac{1}{2}\ln\left((,\frac{1+r_{f}}{1-r_{f}},)\right)$$

The motivation behind such a choice is twofold. Meta‐analytic methods usually assume that the sampling distribution of the observed outcomes is (at least approximately) normal. When the true correlation *ρ* is close to −1 or +1 (and the sample size is small), the sampling distribution of the partial correlation it is skewed and it does no longer well approximate a normal distribution. *Fisher's z‐score* corrects for skewness. Furthermore, to calculate the partial correlation variance (and thus the SEs), the unknown value of *ρ* should be estimated. This can be done by using the observed partial correlation, but, in small samples, this might be a rather inaccurate estimate. On the other hand, the sampling variance of a *Fisher's z‐score* is (approximately) equal to:

(4) $$\mathrm{var}\left(z\right)=\frac{1}{n-3}$$

which no longer depends on unknown quantities.^8^

#### 4.4.3 Unit of analysis issues

Units of analysis are (university) students in most cases (around 70%). Given that students might display a different behavior than other samples of the population, we test whether results are different when students are the unit of analysis in the meta‐regression analysis. Findings of the random effect model with clustered standard errors suggest that studies using students as the unit of analysis find a higher reduction of the level of corruption than studies using subjects other than students. However, such a result is not robust to alternative specification, as well as to the adoption of a multilevel model, which relaxes the assumption imposed by clustered standard errors of within‐cluster homogeneity.

#### 4.4.4 Dealing with missing data

Studies not reporting a measure of dispersion of the collected coefficients (i.e. its standard errors or t‐statistics) are excluded from the analysis due to impossibility of computing the partial correlation coefficient.

#### 4.4.5 Assessment of heterogeneity

The degree of heterogeneity is in line with that found in several other meta‐analyses in economics (Ioannidis et al., 2017). Indeed, the amount of the total variance due to heterogeneity (i.e. the $I^{2}$) is equal to 92.36%. The part due to the between‐study heterogeneity and within‐study heterogeneity is 43.7% and 48.57%, respectively. Meta‐regression models were adapted to include several covariates allowing to investigate the determinants behind the observed heterogeneity.

#### 4.4.6 Assessment of reporting biases

See section on publication bias.

#### 4.4.7 Data synthesis

Meta‐(regression) analysis models are usually divided between fixed effect and random effect(s) models. Recently, Stanley & Doucouliagos, 2017) challenged the conventional meta (regression) models by proposing a third alternative: the Unconditional Weighted Least Square Meta‐Regression Analysis (henceforth UWLS‐MRA).

Conventional meta‐analytic models assume that the sampling variances are known. Conversely, the UWLS‐MRA assumes that the sampling variances are known only up to a proportionality constant $\sigma_{e}^{2}$, which is “automatically estimated by the mean squared error, ${\mathrm{MS}}_{E}$” (Stanley & Doucouliagos, 2017, p. 22). Standard meta‐analytic models instead suppose that $\sigma_{e}^{2}$ = 1. Assuming q predictors to explain between‐studies difference, the UWLS‐MRA can be written as:

$$y_{i}=\theta_{0}+\theta_{1}X_{1i}+\text{⋯}+\theta_{q}X_{\mathrm{qi}}+\varepsilon_{i},\varepsilon_{i}∼N\left(0,\sigma_{e}^{2}v_{i}\right)$$

where$X_{1i}$, …, $X_{\mathrm{qi}}$ are study‐level predictors and $\theta_{1}$, …, $\theta_{q}$ their coefficients. The model is then fitted via weighted least squares with weights $\omega_{i}$ equal to 1∕($\sigma_{e}^{2}v_{i}$). Therefore, while in random effect(s) meta‐analysis unrestricted inferences is obtained through an additive factor (i.e., $\tau^{2}$), the UWLS‐MRA addresses excess heterogeneity via a multiplicative factor. There is little or no rationale for such a multiplicative factor. As Thompson and Sharp report, “[T]he idea that the variance of the estimated effect within each study should be multiplied by some constant has little intuitive appeal, […] we do not recommend them in practice” (Thompson & Sharp, 1999, p. 2705). However, the simulations reported by Stanley & Doucouliagos, 2017) show that there is little difference between random effects models and the UWLS‐MRA, which appears to be even superior when there is a sizeable.

After trying several specifications, we selected the following ten covariates (*q* predictors):

- Students vs other participants to lab. Exp.: illustrates whether the participants of the lab experiment were students or not.
- Low.income: identifies experiments developed in countries with a GNI per capita of $995 or less.
- Low.middle.income: identifies experiments developed in countries with a GNI per capita between $996 and $3,895.
- Upper.middle.income: identifies experiments developed in countries with a GNI per capita between $3,896 and $12,055.
- Control/Deterrence interventions vs Organizationl/Cultural: identifies the macro‐category of anti‐corruption interventions.
- Economic vs other types of intervention: identifies the types of anti‐corruption interventions.
- More than one intervention: identifies experiments where more than one anti‐corruption intervention was developed at the same time.
- Embezzlement vs other types of corruption: identifies the types of corruption.
- Extortionary vs collusive corruption: identifies the macro‐category of corruption.

Quality of the study: identifies the quality of study measured as IDEAS/RePEc Simple Impact Factors (Last 10 Years) for Journals (https://ideas.repec.org/top/top.journals.simple10.html) or IDEAS/RePEc Simple Impact Factors (Last 10 Years) for Working Paper Series (https://ideas.repec.org/top/top.wpseries.simple10.html).

A variable distinguishing between lab‐experiments and field‐experiments was included in previous models but, as far as we found no significant difference between the two populations, and the covariate was not adding any additional information to the model‐the other variables were not changing, we removed it.

#### 4.4.8 Sensitivity analysis

The robustness of the result is tested by adopting different heterogeneity estimators as well as different meta‐analytical models. Overall, findings are largely unchanged.

#### 4.4.9 Deviations from the Protocol

This review entails some deviations from the Protocol published in January 2016 on the Campbell Collaboration library. These changes are reported and justified below.

##### Title of the review

We simplified the title in order for it to be self‐explaining and avoid repetition. In particular, we substituted the term “administrative reforms” with “public sector reforms”.

##### Types of interventions

The Protocol referred to six types of interventions (i.e. Economic, Educational, Cultural, Organisational or bureaucratic, Political, Judicial or repressive). The actual review still consider these anti‐corruption interventions but it groups them in two main categories: 1) Control/Deterrence, and Organizational/Cultural interventions. The choice of focusing on these two categories can be justified from an empirical and theoretical point of view. Indeed, these two domains are able to capture the majority of anti‐corruption interventions considered in the literature (see section on “Intervention” for further details), and, at the same time, allow for a parsimonious statistical model.

Indeed, the initial classification of anti‐corruption interventions (based on six sub‐categories) did not show robust results in the meta‐regression model. Anyway, the effect of the initial categorization is still considered in the actual review by the introduction in the meta‐regression model of the independent variable “Economic vs other type of intervention”.

##### Search strategy

In the Protocol, we initially stated that the literature search would have performed in order to obtain studies written in several languages. In the actual review we restricted our queries to return only studies in English, because we noticed that experimental studies in this field of research are translated into English to ensure their results’ dissemination. No other restriction to the literature search was applied.

Changes in the search strings related to the types of corruption were applied to avoid overlapping results (e.g., diversion of state assets and diversion of state revenue returned the same results in terms of studies, therefore we kept only “diversion and asset”). The deletion of the search strings “integrity” and “misconduct” was applied because the search by these keywords ended up in too generic results and outcomes concerning not only corruption. The deletion of some keywords related to the method of the study (e.g., quasi‐experimental, time‐series, etc.) is due to the fact that we excluded quasi‐experimental studies and we focused only on RCTs.

##### Type of studies eligible for inclusion

In the Protocol, studies eligible for inclusion in the review referred to randomized controlled trials (RCTs), natural experiments, interrupted time‐series designs, and any other quasi‐experimental design with or without a control group (e.g. pretest‐posttest two groups studies and pretest‐posttest one group studies), as well as cross‐sectional studies like surveys. In the actual review, we exclude quasi‐experimental studies and focus on RCTs only. Indeed, the absence of proper randomization could undermine the validity of quasi experiment and natural experiment. With quasi‐natural experiments, randomization is only true as long as you believe the identification strategy proposed by the author(s). There is now substantial evidence that RCTs are the least biased type of study available in social sciences. Brodeur et al. (2018) show that selective publication and p‐hacking is a substantial problem in research employing DID and (in particular) IV (which are quasi‐natural experiments). RCT and RDD are much less problematic. Further, in our context, quasi‐natural experimental are much more linked to the replication crisis than lab‐experiments with students as participants. Indeed, the former gives to the author(s) several degrees of freedom of research (which can lead to p‐hacking), while the latter can have, at worst, low or null external validity.

When it comes to study causal relationships, experimental studies are known to be the more valid ones. Randomization allows to control for any potential confounder and RCTs allow to make causal inference, whereas observational studies cannot. Furthermore, including other types of empirical studies would have likely increased the between‐study heterogeneity, making our conclusion somehow less reliable, as studies included in a meta‐analysis should share similar underlying characteristics. Finally, if more qualitative studies would have been included, we could have not performed a meta‐analysis.

## 5 RESULTS

### 5.1 Description of studies

#### 5.1.1 Results of the search

A total of seventy studies have been identified by this systematic review. The initial literature search, conducted between April and August 2018, led to the identification of sixty‐one studies. An additional literature search developed in May 2020 led to the identification of nine additional studies. Approximately one‐third of them were excluded at the title/abstract stage because they did not evaluate any anti‐corruption intervention but simply assessed the relationship between corruption and other phenomena. Or because the study design was not based on randomized controlled trials. Other fourteen studies were excluded only after a full‐text assessment. At this stage, the main reasons for exclusion were related to an unsuitable type of outcome (corruption),^9^ unsuitable measurement of the outcome,^10^ the lack of regression output and unsuitable study design.

At the end of the selection process twenty‐nine studies resulted as eligible for inclusion.

The flow chart below (Figure 4) illustrates the inclusion/exclusion process of studies in detail.

**Figure 3:**
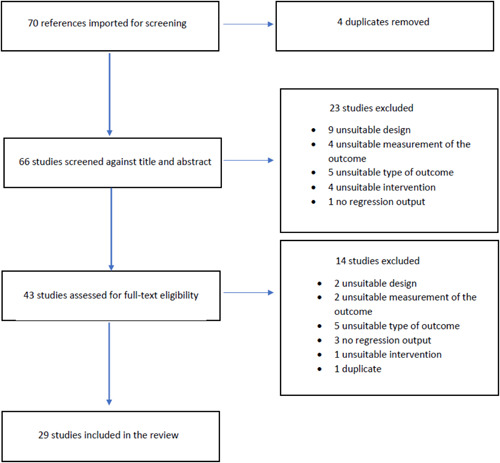
Stages of the literature search

**Figure 4:**
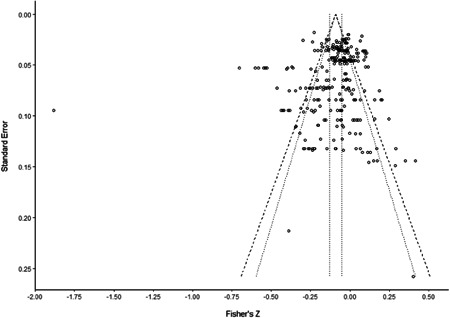
Funnel plot for publication bias

#### 5.1.2 Included studies

All the selected studies are written in English. They span the period from 2007 to 2018 and cover 16 different countries. . The majority of the selected studies (twenty) investigate the effect of anti‐corruption interventions in high and upper‐middle income countries, namely Austria, Brazil, Canada, China, Germany, Italy, Mexico, the Netherlands, Thailand, the United Kingdom, and the United States. Nine studies cover low and low‐middle income countries, namely Burkina Faso, Burundi, Ethiopia, India, Indonesia, Tanzania, and Uganda. All studies are randomized controlled trials, with 25 conducted in a laboratory, while four are field experiments (see Table 2 below).

**Table 2:** Characteristic of included studies

N	Author name (year)	Study design (country)	N. units of analysis	Main intervention (type of intervention)	Type of corruption (proxy)	Sector
1	Ryvkin et al., (2017)	Lab experiment (US)	163	Control/Deterrence (economic)	Extortionary (passive and active bribery)	Public administration
2	Banerjee et al. (2016)	Field experiment, (India)	1397	Organizational/cultural (organizational)	Extortionary (embezzlement)	Public administration
3	Buckenmaier et al. (2018)	Lab experiment (Italy)	268	Organizational/cultural (cultural)	Collusive (active bribery)	Public administration
4	Ryvkin and Serra (2017)	Lab experiment (US)	216	Organizational/cultural (organizational)	Extortionary (both passive and active bribery)	Public administration
5	Songchoo and Suriya (2012)	Lab experiment (Thailand)	48	Control/Deterrence (economic)	Collusive (embezzlement)	Public administration
6	Bigoni et al. (2015)	Lab experiment (Italy)	256	Control/Deterrence (economic) Organizational/cultural (cultural)	Collusive (embezzlement)	Public administration
7	Campos‐Vazquez and Mejia (2016)	Lab experiment (Mexico)	164	Control/Deterrence (economic)	Collusive (both passive and active bribery)	Public administration
8	Nyqvist, de Walque and Svensson (2017)	Field experiment (Uganda)	3750	Organizational/cultural (organizational)	Extortionary (embezzlement)	Health sector
9	Abbink et al. (2014)	Lab experiment (India)	360	Control/Deterrence (legal)	Extortionary (passive bribery)	Public administration
10	Banerjee, R., & Mitra, A. (2018)	Lab experiment (India)	258	Control/Deterrence (mixed) Organizational/cultural (educational)	Extortionary (passive bribery)	Public administration
11	Serra (2011)	Lab experiment (UK)	180	Control/Deterrence (organizational control)	Collusive (passive bribery)	Public administration
12	Olken (2007)	Field experiment (indonesia)	608	Control/Deterrence (organizational control)	Collusive (embezzlement)	Public construction
13	Abbink and Wu (2017)	Lab experiment (China)	198	Control/Deterrence (economic)	Collusive (active bribery)	Public administration
14	Van Veldhuizen (2013)	Lab experiment (Netherlands)	76	Control/Deterrence (economic)	Collusive (passive bribery) Collusive (favoritism)	Public administration
15	Barr et al. (2009)	Lab experiment (Ethiopia)	144	Control/Deterrence (organizational control) Control/Deterrence (economic) Organizational/cultural (cultural)	Extortionary (embezzlement)	Health sector
16	Christofl, Leopold‐Wildburger and Rasmußen (2017)	Lab experiment (Austria)	180	Control/Deterrence (organizational control) Organizational/cultural (cultural) Control/Deterrence (mixed)	Collusive (active bribery)	Public administration
17	Falisse and Leszczynska (2015)	Lab experiment (Burundi)	527	Organizational/cultural (cultural)	Collusive (both passive bribery and favoritism)	Public administration
18	Banuri and Eckel (2015)	Lab experiment (US/Pakistan)	109	Control/Deterrence (economic)	Collusive (both active bribery and favoritism)	Public administration
19	Azfar and Nelson (2007)	Lab experiment (US)	96	Control/Deterrence (economic) Organizational/cultural (organizational) Organizational/cultural (educational)	Extortionary (embezzlement)	Public administration
20	Zamboni and Litschig (2018)	Field experiment (Brazil)	5520	Control/Deterrence (organizational control)	Extortionary (both embezzlement and favoritism)	Public administration
21	Ryvkin and Serra (2016)	Lab experiment (US)	136	Organizational/cultural (economic) Organizational/cultural (organizational)	Extortionary (passive and active bribery)	Public administration
22	Engel et al. (2016)	Lab experiment (Germany and China)	96	Control/Deterrence (legal)	Collusive (active bribery)	Public administration
23	Di Falco et al. (2016)	Lab experiment (Tanzania)	1080	Organizational/cultural (organizational)	Extortionary (embezzlement)	
24	Makowsky and Wang (2018)	Lab experiment (US)	216	Organizational/cultural (organizational)	Extortionary (embezzlement)	Public administration
25	Khachatryan et al.(2015).	Lab experiment (Germany)	96	Control/Deterrence (organizational control)	Extortionary (both active and passive bribery)	Public administration
26	Khadjavi et al. (2017)	Lab experiment (Germany)	356	Control/Deterrence (organizational control)	Extortionary (embezzlement)	Public administration
27	Salmon and Serra (2017)	Lab experiment (US)	432	Organizational/cultural (educational)	Collusive (active bribery)	Public administration
28	Armantier and Boly (2008)	Lab experiment/field experiment (Canada)	145	Control/Deterrence (economic) Control/Deterrence (organizational control)	Collusive (passive bribery)	Education
29	Armantier and Boly (2014).	Lab experiment (Burkina Faso)	97	Control/Deterrence (economic)	Collusive (passive bribery)	Education

Concerning the type of outcome, the majority – eighteen ‐ of the selected studies address bribery (either active or passive), while eleven studies consider misappropriation of public resources (embezzlement). In terms of anti‐corruption interventions, nineteen studies test the effect of control and deterrence interventions while ten studies are focused on organizational/cultural interventions.

Among those studies that tested the effect of organizational/cultural interventions, the field experiment of Björkman Nyqvist et al. (2017) is the most recent one. The authors tested the longer run impact of a local accountability intervention in primary health care provision in Uganda, and in particular on issues such as absenteeism. In particular, two interventions were developed in 2005 and evaluated in 2007 and 2008: “*participation”* and “*participation & information*”. The *participation* intervention involved three types of meetings facilitated by staff from local community‐based organizations. The main objective of these meetings was to encourage community members and health facility staff to develop a shared view of how to improve service delivery and monitor health provision in the community. The *information & participation* intervention also entailed meetings between community members and the health facility employees. In this case, before the meetings took place, the participants were provided with easily accessible quantitative data on the performance of the health provider. These data were collected from facility and household surveys implemented prior to the intervention. Sharing this data allowed to address informational asymmetries between the providers and beneficiaries and to guide the discussion towards issues that potentially could be dealt with locally. The authors found that the intervention focused only on increasing participation, but not reducing possible informational asymmetries, had little impact, while the *information & participation* intervention resulted in “a more engaged community and in large and long‐run improvements in both health service provision and health outcomes” (Björkman Nyqvist et al., 2017: 36).

With regard to studies testing the effect of control and deterrence interventions, Zamboni and Litschig (2018) conducted a randomized policy experiment to test whether increased audit risk deters rent extraction in three areas of local government activity in Brazil: procurement, health service delivery and cash transfer targeting. The annual audit risk was increased from a baseline level of about 5 percent to about 25 percent across the experimental group of local government activities. Their results suggest that “increasing annual audit risk by about 20 percentage points reduced the share of audited resources involved in corruption in procurement by about 10 percentage points and the proportion of procurement processes with evidence of corruption by about 15 percentage points” (Zamboni & Litschig, 2018: 133). They also found that their results are invariant to alternative corruption definitions. In contrast, they found that the increased audit risk did not affect the quality of publicly provided preventive and primary health care services or compliance with eligibility requirements for the conditional cash transfer program. The authors argued that the different impacts of audit risk in procurement vs. health service delivery is mainly due to the seriousness of the potential punishment/sanctions and the probability that a sanction is applied. Indeed, “for service delivery irregularities, sanctions include at most the loss of the job. For public officials in charge of procurement in contrast, potential sanctions are relatively high as they include not only job termination but also fines as well as jail time” (Zamboni & Litschig, 2018: 135). Similarly, “The sanctioning probability in service delivery is likely low because irregularities in service provision cannot be unambiguously identified through an audit. … In contrast, irregularities in procurement are relatively easy to prove because local officials are required to document each step of the purchasing process” (Zamboni & Litschig, 2018: 135).

A summary of all included studies is reported in the Annex.

#### 5.1.3 Excluded studies

We excluded forty‐one studies. Twenty‐seven at the title/abstract stage and fourteen studies after a full‐text assessment.

Table 3 below summarizes the reasons for the exclusion of these reports. References of the excluded papers are available in the dedicated section.

**Table 3:** Synthesis of the reasons for exclusion of 41 papers

Reason for exclusion	N	%
Unsuitable design (no RCTs)	11	27%
Unsuitable measurement of the outcome (no experience of corruption)	6	15%
Unsuitable type of outcome (no administrative corruption)	10	24%
No regression output	4	10%
Unsuitable intervention	5	12%
Reports based on the same data (duplicate)	5	12%

##### Unsuitable design

We excluded 11 studies (27%) because they were not based on randomized control trials (RCTs) but rather on natural experiments (e.g., Asthana 2009; Borcan et al., 2014; Celiku, 2013) or other types of design (e.g., DiTella & Schargrodsky, 2003; Durante et al., 2011; Dusek et al. 2015; etc.). We excluded the systematic review of Molina et al., 2016 because it does not include RCTs only but also quasi‐experimental studies. However, we used it as source for identifying primary research reports.

##### Unsuitable type of outcome

We excluded 10 studies (24%) because their dependent variable (outcome) was not administrative corruption. Lierl (2017) focuses on political corruption; Peisakhin & Pinto, 2010 define corruption as access to service; Belot (2013) defines corruption as theft from students who volunteer to do a certain job. Murray (2017) considers corruption as back‐scratching (emergence of alliances between players). The study of Chen (2009) refers to private‐to‐private corruption.

##### Unsuitable measurement of the outcome

We excluded 6 studies (15%) because they did not measure experiences of corruption but rather perceptions of corruption (e.g., Denisova‐Schmidt 2015; Tavares, 2007), or willingness to engage in corrupt practices (Corbacho et al., 2016). In Tavares (2007) corruption was measured by the International Country Risk Guide (ICRG) index, and by the Transparency International Corruption Perception Index (TI CPI), as well as in Denisova‐Schmidt (2015). Corbacho et al. (2016) evaluate the impact of information on the willingness to bribe a police officer.

##### Unsuitable regression output

We excluded 4 studies (10%) because they did not include suitable regression output (e.g., Feess et al., 2018; Wang, 2009) or quantitative outcome (e.g., Zhang, 2015).

##### Unsuitable intervention

We excluded 5 studies (12%) because the tested intervention was not aimed at reducing administrative corruption (e.g., Banerjee, 2016; Djawadi & Fahr, 2013; Drugov et al., 2012; Gaggero 2018).

### 5.2 Risk of bias in included studies

#### 5.2.1 Risk of bias

The risks of bias of included studies was initially assessed using the Cochrane Risk of Bias checklist as a basis.

During the screening of the included studies, it emerged that the most important elements to be taken into consideration for evaluating the risk of bias were the internal and external validity. Indeed, it emerged that not all the criteria considered in the Cochrane Risk of Bias checklist were observable and/or applicable to our selected studies. Those criteria have been created to address studies in the medical sector while our review includes social‐economic papers. Furthermore, as far as our review includes only randomized controlled trials or lab experiments presenting some sort of randomization, the internal validity of selected studies is supposed to be high and many elements of the Cochrane's checklist became, thus, not relevant. Issues of external validity of our selected studies are mainly related to the representativeness of the samples.

We define internal validity as the degree of confidence that the identified relationship is not affected by other variables (i.e., confounding factors), endogeneity and\or other model misspecifications. In other words, the identification strategy is, to some extent, correct.

We consider external validity as the possibility to generalize the inference obtained from the identified relationship to the whole population (e.g., to what extent results obtained with students in a lab could be generalized to the population of interest?).

Table 4 below shows the internal and external validity assessed for each included study.

**Table 4:** Risk of bias on included studies

study	author	internal.validity.score	internal.validity.comment	external.validity.score	external.validity.comment
20	Ryvkin, Serra, and Tremewan (2017)	high	NA	high/low	The author states that: “There is substantial evidence of the external validity of qualitative results generated by lab experiments (Camerer, 2011; Kessler and Vesterlund, 2011). With respect to corruption, Armantier and Boly (2013) have shown that corruption “can be studied in the lab.”.
55	Banerjee et al. (2016)	high	NA	high/high	It is a field experiment.
54	Buckenmaier et al. (2018)	high	NA	low/low	They never mention it and it is a lab experiment with undergraduate students.
47	Ryvkin and Serra (2017)	high	NA	high/low	They wrote that: "It may be argued that our results have little external validity as they are generated by incentivized games played with university students in a developed country characterized by low corruption. However, previous research on corruption (Armantier and Boly, 2013; Barr and Serra, 2010) has shown that results generated by lab experiments in developed countries are informative about actual corruption and responsiveness to incentives in developing countries. Moreover, there is widespread consensus on the external validity of the qualitative results generated by laboratory experiments addressing policy‐research questions.".
43	Songchoo and Suriya (2012)	low	No detailed description of how the experiment was conducted	low/low	It is a lab experiment with no details about external validity.
32	Bigoni et al. (2015)	high	NA	low/low	They never mention external validity and it is a lab experiment with undergraduate students.
30	Campos‐Vazquez and Mejia (2016)	high	NA	low/low	It is a lab experiment with undergraduate students. Furthermore, the authors simply mention that: “Additional research is needed on the external validity of previous findings as applied to developing countries”.
29	Björkman Nyqvist et al. (2017)	high	NA	high/high	It is a field experiment.
2	Abbink et al. (2014)	high	NA	high/low	The authors mention that: "The use of context‐specific instructions also improves the external validity of our results".
4	Banerjee R. and Mitra, A (2018)	high	NA	high/low	The authors state that: "studies show that results obtained through laboratory experiments, particularly laboratory corruption games, are externally valid and they do measure moral cost of engaging in corruption (Armantier and Boly, 2013; Banerjee, 2015)".
11	Serra, D. (2012)	high	NA	high/low	The author states that: "external validity is open to debate", but quote a study by Barr and Serra to support the validity of her experiment (pp. 4‐5).
12	Olken, B. (2005)	high	NA	low/low	
21	Abbink and Wu (2017)	high	Na	low/low	
23	Van Veldhuizen (2013)	high	NA	high/low	Actually the author quotes the study by Armantier and Boly (in favour of the validity of lab experiments) but the author also recognises that: "to what extent these findings will generalize to the field settings and other subject pools remains an open question.".
26	Barr et al. (2009)	high	NA	high/high	The authors mention that: "The subjects were sampled by, first, randomly selecting one government, one NGO, and one private nursing school and then randomly selecting students from within each school. Students could refuse to participate, but refusals were rare and there was no apparent pattern in the reason given”.
41	Christofl, A., Leopold‐Wildburger, U., Rasmuen, A	high	NA	high/low	The authors report that their framing increases external validity.
42	Falisse and Leszczynska (2015)	high	NA	high/high	The authors mention that: "…key methodological challenge is to find ways to articulate the advantages of laboratory and field research in producing contributions that can claim some level of external validity. These concerns are especially relevant in the present study, which is primarily concerned with the identity of the participants … Hence our decision to organize a lab‐in‐the‐field,. . where the selected participants are also the subjects of our study, i.e., public servants".
46	Banuri and Eckel (2015)	high	NA	high/low	Authors state that:" Perhaps the strongest critique of lab studies in this arena is its weak external validity, particularly when using developed‐country subjects. We attempt to account for this potential shortcoming by using subjects from two countries.".
49	Schulze and Björn (2003)	high	NA	low/low	
53	Azfar and Nelson (2007)	high	NA	low/low	
58	Litschig, S. and Zamboni, Y.(2018)	high	NA	high/high	Authors state that: “While additional studies are required to assess the external validity of our findings, we believe that many of the key features of the Brazilian setting ‐ (…) are common in many other settings and so our results might be fairly general”.
3	Ryvkin, D. and Serra, D.	high	NA	high/low	The authors quote Barr and Serra (2010) and Armantier and Boly (2013) (see footnote 7, p. 4).
9	Engel et al. (2016)	high	NA	high/high	
15	Di Falco et al. (2016)	high	NA	low/low	
22	Khachatryan et al.(2015)	high	NA	low/low	
19	Salmon and Serra (2017)	high	NA	hihg/low	Footnote 5 There is substantial evidence of the external validity of qualitative results generated by lab experiments (Camerer, 2011; Kessler and Vesterlund, 2011).
13	Abbink et al., 2002	high	NA	high/low	Footnote n 2

**Table 5:** Publication bias tests

		beta	se	T	ci (low)	ci (up)
*Multiplicative*	Intercept	−0.038	0.029	−1.316	−0.094	0.019
	SE	−0.871	0.616	−1.416	−2.079	0.335
*Additive*	Intercept	−0.073*	0.033	−2.180	−0.142	−0.004
	SE	−0.292	0.485	−0.603	−1.288	0.033

Egger et al., (1997) and Stanley (2008). Additive refers to a funnel plot asymmetry test using an additive dispersion term – see Egger and Sterne (2005). Beta is the estimated coefficient; se its standard errors; t the t‐statistic; ci (low) and ci (up) are, respectively, the lower and upper bounds of the confidence interval. Significance codes: p < 0.001 '***' p < 0.01 '**' p < 0.05 '*'

**Table 6:** Results of the meta‐analysis models

	beta	se	z	ci (low)	ci (up)
*RE*	−0.091	0.010	−9.382	−0.110	−0.072
*RE (cluster)*	−0.091	0.020	−4.451	−0.133	−0.049
*REs*	−0.074	0.023	−3.290	−0.118	−0.030

Beta is the estimated coefficient; se its standard errors; z the z‐statistic; ci (low) and ci (up) are, respectively, the lower and upper bounds of the confidence interval.

#### 5.2.2 Publication bias

**Figure 5:**
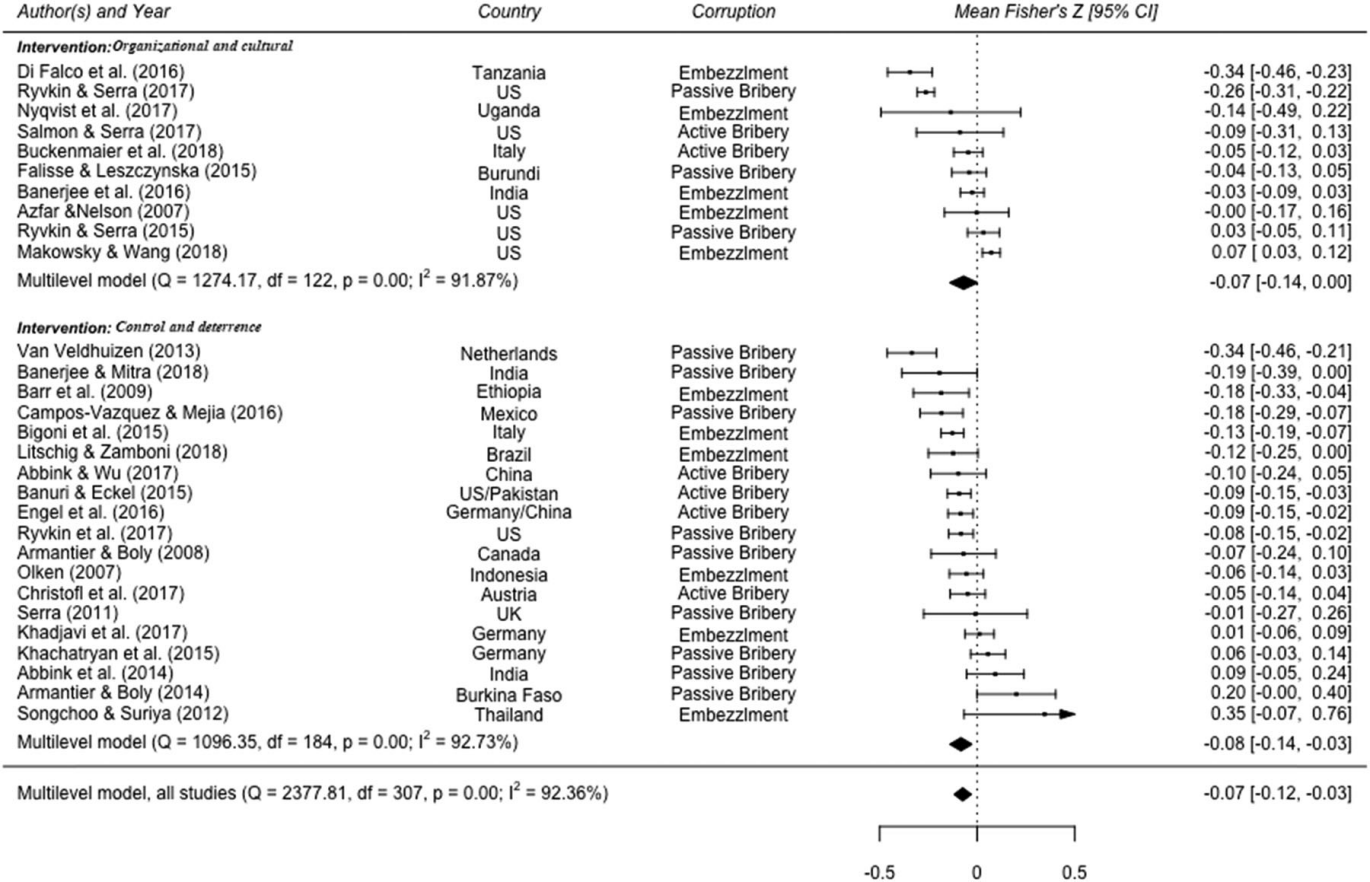
Forest plot by type of intervention and corruption

The most common way of assessing publication bias is the visual investigation of funnel plots. The latter are scatter plots of the effect size estimates from individual studies against an inverted measure of the study size ‐ usually the standard error. Contrary to the conventional graphical displays for scatter plots, funnel plots present point estimates on the horizontal axis and study size on the vertical axis (see Figure 5 below).

Theoretically, estimates with a smaller sample size should produce less precise effect sizes. As the sample size increases, the size of the standard errors decreases. Therefore, the graph should resemble an inverted funnel; low‐sample studies should scatter more widely at the bottom, while large‐sample researches should be less dispersed around the underlying true effect size. Asymmetry of the scatter plot is said to be a sign of publication bias; if small studies that are unable to reject the null hypothesis are either suppressed or not accepted for publication there would be a gap in one of the graph's bottom corners. Conversely, a symmetric inverted funnel shape implies no publication bias.

In order, to test whether there is asymmetry, Egger's tests with additive and multiplicative dispersion terms were used. There is no evidence of a substantial publication bias in the literature.

### 5.3 Synthesis of results

#### 5.3.1 Effect sizes

The forest plot below illustrates the average value of the findings of each study, by type of intervention and corruption. Each horizontal line represents an individual study with the result plotted as a box and the 95% confidence interval of the result displayed as the line. When a study crosses the vertical line (the line of null effect), this indicates that the null value lies within the 95% confidence interval. Therefore, the mean of the different findings (e.g., concerning different interventions and/or types of corruption) of that specific study cannot reject the null effect of the intervention.

For example, concerning organizational and cultural interventions, the findings of Di Falco et al. (2016) show that, on average, in Tanzania making information more transparent and reducing the number of intermediaries in transfer chains can reduce embezzlement, and that this negative impact is statistically significant. On the other side, the average findings of Salmon and Serra (2017) did not observe a statistically significant impact of social observability and the possibility of social judgment on active bribery in the United States.

Concerning control and deterrence interventions, the study of Van Veldhuizen (2013) reports a significant effect of higher public officials’ wages in combination with monitoring in reducing passive bribery. While the findings of Abbink and Wu (2017) on the effect of offering a reward for self‐reporting to reduce active bribery are not statistically significant.

Overall, the multilevel (random effects) meta‐analysis indicates that the identified interventions decrease the level of corruption. Such result is statistically significant (p < 0.01). The high degree of heterogeneity (I^2^ = 92.36%) of the studies, even if in line with that found in several other meta‐analyses in economics, suggests the need for a meta‐regression analysis (see paragraph below).

#### 5.3.2 Meta‐analysis

Random effect(s) models have been preferred to fixed effect models for this review, due to the nature of the data (i.e., presence of multiple effect sizes per study).

The results of the random effect model (RE), the random effect model with clustered standard errors (RE cluster), and the multilevel (random effects) model (REs), show an overall (and highly comparable) negative effect. This indicates that the recorded interventions reduce the level of corruption.^11^

Overall, the degree of heterogeneity is in line with that found in several other meta‐analyses in economics (Ioannidis et al., 2017), as the $I^{2}$ is 92.36%. The multilevel model show that the part due to between‐study heterogeneity and within‐study heterogeneity is, respectively, 43.79% and 48.57%.

#### 5.3.3 Meta‐regression analysis

Meta‐regression models were adopted to investigate the potential determinants of the aforementioned between‐ and within‐study heterogeneity.

The results of the random effect model (Table 7) suggest that:

1) The interventions included in the selected studies reduce the level of corruption more in low‐income and upper‐middle income countries than in high‐income countries.
2) Studies conducted using a combination of different interventions show a higher reduction of corruption than studies conducted using only one type of intervention.

**Table 7:** Random effect Model, clustered standard error

Model results	beta	SE	z	p value	ci (low)	ci (up)
*Students vs other participants lab. Exp*.	−0.1007.	0.0492	−2.0457	0.0557	−0.2042	0.0027
*Low.income*	−0.1496*	0.0604	−2.4768	0.0234	−0.2764	−0.0227
*Low.middle.income*	0.0010	0.0396	0.0253	0.9801	−0.0821	0.0841
*Upper.middle.income*	−0.0840.	0.0480	−1.7490	0.0973	−0.1849	0.0169
*Control/Deterrence vs Organizational/Cultural interventions*	0.0034	0.0337	0.1016	0.9202	−0.0674	0.0743
*Economic vs other types interventions*	−0.0423	0.0369	−1.1478	0.2661	−0.1198	0.0351
*More than one intervention*	−0.2386**	0.0664	−3.5934	0.0021	−0.3781	−0.0991
*Embezzlement vs other types of corruption*	−0.0454	0.0324	−1.4011	0.1782	−0.1135	0.0227
*Extortionary vs collusive corruption*	0.0227	0.0277	0.8189	0.4236	−0.0355	0.0809
*Quality of the study*	−0.0041.	0.0022	−1.8290	0.0840	−0.0088	0.0006
*Intercept*	0.0737	0.0558	1.3211	0.2030	−0.0435	0.1908

beta is the estimated coefficient; se its standard errors; z the z‐statistic; p value the estimated p value. ci (low) and ci (up) are, respectively, the lower and upper bounds of the confidence interval. There are 308 effect sizes nested around 29 papers, with a mean of 10.62 effect sizes per study, a median of 8, a minimum of 2 and a maximum of 32. Significance codes: p < 0.001 '***' p < 0.01 '**' p < 0.05 '*'.

The results of the multilevel (random effects) model (Table 8) show that:

1) Control and deterrence interventions are more effective than organizational and cultural interventions in reducing administrative corruption;
2) Studies conducted using a combination of different interventions show a higher reduction of corruption than studies conducted using single interventions. This result is robust across all models.
3) The identified interventions are more effective in reducing the level of misappropriation of public resources (embezzlement) than passive or active bribery.

**Table 8:** Multilevel Meta‐Analysis Model

Model results	beta	SE	z	p value	ci (low)	ci (up)
*Students vs other participants lab. Exp*.	−0.0814	0.0944	−0.8627	0.3883	−0.2663	0.1035
*Low.income*	−0.0669	0.0911	−0.7342	0.4629	−0.2455	0.1117
*Low.middle.income*	0.0828	0.0727	1.1402	0.2542	−0.0596	0.2253
*Upper.middle.income*	0.0390	0.0716	0.5449	0.5858	−0.1013	0.1793
*Control/Deterrence vs Organizational/Cultural interventions*	−0.1131**	0.0425	−2.6611	0.0078	−0.1965	−0.0298
*Economic vs other types interventions*	−0.0098	0.0390	−0.2514	0.8015	−0.0862	0.0666
*More than one intervention*	−0.1337*	0.0522	−2.5592	0.0105	−0.2360	−0.0313
*Embezzlement vs other types of corruption*	−0.1084*	0.0476	−2.2750	0.0229	−0.2018	−0.0150
*Extortionary vs collusive corruption*	0.0165	0.0657	0.2512	0.8017	−0.1123	0.1453
*Quality of the study*	−0.0030	0.0062	−0.4771	0.6333	−0.0152	0.0093
*Intercept*	0.1088	0.1073	1.0146	0.3103	−0.1014	0.3191

beta is the estimated coefficient; se its standard errors; z the z‐statistic; p value the estimated p value. ci (low) and ci (up) are, respectively, the lower and upper bounds of the confidence interval. There are 308 effect sizes nested around 29 papers, with a mean of 10.62 effect sizes per study, a median of 8, a minimum of 2 and a maximum of 32. Significance codes: p < 0.001 '***' p < 0.01 '**' p < 0.05 '*'.

We also tested whether there is any difference between laboratory and field experiments by introducing a dummy variable distinguishing between them in the multilevel model. Results show no difference between laboratory and field experiments (a result already known in the literature, see for example Armantier & Boly, 2011; Camerer 2012; Whang 2009), as the coefficient associated with the dummy variable is never statistically different from zero. However, such a result could be driven by power‐issues as the field experiments in our sample are 4 out of 29. Therefore, in Table 9 we investigate whether a sample containing laboratory experiments only provides different results than those shown in Table 8.

**Table 9:** Multilevel Meta‐Analysis Model: laboratory‐based studies only

Model results	beta	SE	z	p value	ci (low)	ci (up)
*Students vs other participants lab. Exp*.	−0.2532	0.1344	−1.8831	0.0597	−0.5167	0.0103
*Low.income*	−0.2	0.1067	−1.8751	0.0608	−0.409	0.009
*Low.middle.income*	0.1036	0.0889	1.1654	0.2439	−0.0707	0.2779
*Upper.middle.income*	0.0817	0.0857	0.9537	0.3402	−0.0863	0.2497
*Control/Deterrence vs Organizational/Cultural interventions*	−0.0978*	0.048	−2.0381	0.0415	−0.1918	−0.0038
*Economic vs other types interventions*	−0.0165	0.0453	−0.3652	0.715	−0.1053	0.0722
*More than one intervention*	−0.1461*	0.0599	−2.4382	0.0148	−0.2635	−0.0287
*Embezzlement vs other types of corruption*	0.0545	0.0846	0.6441	0.5195	−0.1113	0.2202
*Extortionary vs collusive corruption*	0.0312	0.0869	0.3586	0.7199	−0.1391	0.2014
*Quality of the study*	−0.002	0.0128	−0.1558	0.8762	−0.027	0.0231
*Intercept*	−0.002	0.0128	−0.1558	0.8762	−0.027	0.0231

beta is the estimated coefficient; se its standard errors; z the z‐statistic; p value the estimated p value. ci (low) and ci (up) are, respectively, the lower and upper bounds of the confidence interval. There are 250 effect sizes nested around 25 papers. Significance codes: p < 0.001 '***' p < 0.01 '**' p < 0.05 '*'.

Overall, results are largely unchanged, albeit the coefficient associated with “Embezzlement vs other types of corruption” is no longer statistically different t0 zero. This could be driven by the fact that of the 96 effect sizes in which embezzlement is the proxy of corruption, *only* 58 are part of laboratory experiments, while the remaining 38 are shown in field experiments.

In addition, we also run a random effect model including only the four identified field experiments. The results of this model still show a negative effect of interventions on corruption, and corroborate the results of the analysis including all twenty‐nine studies. However, the results of this model are statistically unreliable mainly due to the scarce number of field studies (see section “Data and Analyses”, analysis 10 and 11 for further details).

## 6 DISCUSSION

Several anti‐corruption programs have been developed in the past decades across high‐, medium‐ and low‐income countries. However, there is still a lack of empirical research evaluating the effects of these policies.

This systematic review addresses this gap of knowledge by summarizing global information about the effectiveness of anti‐corruption policies developed in the public sector.

### 6.1 Summary of main results

Twenty‐nine experimental studies have been identified, coded and analyzed within this review. Among them, there is a prevalence of laboratory experiment over field experiments (25 out of 29). The effect sizes included in the selected studies have been synthetized through a meta‐regression analysis.

The majority of the selected studies (twenty) analyze the effect of anti‐corruption interventions in high‐ and upper‐middle income countries. Eighteen of them address bribery (either active or passive), while eleven studies consider misappropriation of public resources (embezzlement). In terms of anti‐corruption interventions, nineteen studies test the effect of control and deterrence interventions while ten studies focus on policies based on organizational and cultural change.

Among the latter, we found a strong prevalence of initiatives aimed at increasing transparency of information and at reducing the asymmetry of information available for both suppliers and consumers of public services. Introducing competition in public service delivery is addressed by two studies and proves to be effective in reducing extortionary bribery, but only if the costs that citizens have to bear for searching alternative providers for the same service can be reduced (e.g., lowering transportation costs or improving information sharing mechanisms) (Ryvkin & Serra, 2017).

Among those studies that evaluate control and deterrence interventions, the majority (seven) tested the effect of public officials’ wages on the likelihood of accepting/demanding bribes. Their results are contradictory. While four studies demonstrate that increasing public officials’ wages greatly reduces their corruptibility (Armantier & Boly, 2008; Azfar & Nelson, 2007; Songchoo & Suriya, 2012; Van Veldhuizen, 2013), others find no robust results (Banerjee & Mitra, 2018, Barr et al., 2009). According to the study of Armantier and Boly (2014), salary schemes that combine bonuses with penalties tend to increase officials’ propensity to take bribes, because this type of intervention diverts subjects away from their sense of duty and their ethical responsibilities, thereby lowering the moral cost of corruption.

Five studies test the effect of policies guaranteeing impunity to officials or citizens who report corrupt practices (principal witness/leniency treatment/asymmetric liability) on collusive bribery (Abbink & Wu, 2017; Bigoni et al., 2015; Engel et al., 2016; Christofl et al. 2017; Buckenmaier et al., 2018), and extortionary bribery (Abbink et al., 2014).

Although leniency policies, aimed at encouraging reporting of corrupt practices by guaranteeing impunity to officials or citizens, should discourage demanding bribes in anticipation of the whistleblowing, results of existing studies do not unequivocally support such assumption. In some cases, leniency policies reduce collusive bribery (Bigoni et al., 2015; Buckenmaier et al., 2018) but at the cost of making bribery more profitable for bidders (Christofl et al. 2017). According to one study, leniency does not show a significant impact on collusive bribery alone but it looks promising when associated with policies that limit agent's and client's exposure to one another, such as staff rotation (Abbink & Wu, 2017). In another case, leniency even worsens the situation by increasing the probability of bribery (Engel et al., 2016). With regard to extortionary corruption, the study of Abbink et al. (2014) shows that, when impunity is guaranteed to bribe‐givers, reporting increases and demands for bribes decrease. This effect persists even when bribes were not refunded after the prosecution, showing that intrinsic motivation was the main driver of citizens' reporting behavior. However, this study also shows that retaliation can significantly dampen the effects of the asymmetric treatment. Hence, leniency policies should be implemented along with complementary measures to protect citizens from retaliation.

The review also identifies a large number of studies focused on monitoring interventions (Olken, 2007; Serra 2012; Campos‐Vazquez & Mejia, 2016; Khachatryan et al., 2015; Salmon & Serra, 2017; Zamboni & Litschig, 2018). In particular, both top‐down (e.g., monitoring by managers) and bottom‐up approach (e.g., monitoring by citizens) have been addressed. The majority of the included studies highlight that monitoring interventions are effective in reducing corruption when they are combined between each other (i.e., top‐down and bottom‐up monitoring) or to other types of interventions (e.g., transparency of information). Interestingly, Zamboni and Litschig (2018) demonstrate that the impact of audit risk on corruption depends on the seriousness of potential sanctions and the probability that a sanction is applied.

The multilevel meta‐regression model indicates that control and deterrence interventions are more effective than policies based on organizational and cultural change in curbing corruption in the public sector. Furthermore, the combination of different types of interventions is more effective than single interventions. This result is robust across all models.

In addition, it appears that the identified interventions are more effective in reducing the level of misappropriation of public resources (embezzlement) than passive or active bribery. No robust results are available on the distinction between passive and active bribery and collusive and extortionary corruption.

Furthermore, no significant differences in the effect of anti‐corruption policies emerge between different sectors (i.e.; public administration, health, education, public construction), as well as between studies conducted in the laboratory and field experiments.

Based on the abovementioned analysis and combination of lab and field experiments, this systematic review shows that control and deterrence‐based measures are more efficient, in curbing corruption in the public sector, than interventions based on organizational and cultural change. However, this result might be due to the fact that the majority of selected studies are based on lab‐experiments, where the assessment of the intervention is almost concurrent to its development. Short‐term evaluations might fail to identify the effect of organizational and cultural interventions. Indeed, these interventions are based on structural changes in the organization of the system and the ethical and cultural education of public officials and might, thus, entail long periods to display their results on the level of corruption. Also, in the case of field experiments, the evaluation of the anti‐corruption policies might have been too short. For example, in the study of Björkman Nyqvist et al., 2017, the interventions were developed in 2005 and evaluated in 2007 and 2008.

### 6.2 Overall completeness and applicability of evidence

This systematic search was conducted across a comprehensive set of databases, websites and grey literature sources. Reference harvesting was also conducted to ensure completeness. We included only studies written in or translated into English. This choice was supported by the fact that experimental studies in this field of research are usually translated into English to ensure the dissemination of their results.

The final set of twenty‐nine eligible studies allows an analysis of the effect of control and deterrence interventions, and organizational and cultural policies on corruption in the public sector. This set of studies meets our methodological and substantive criteria and includes only high‐quality experimental designs. It is important to recognize that the majority of included studies are laboratory experiments. Even if the evidence suggests that the external validity and generalizability of the results of laboratory experiments on corruption are similar to the outcomes of field experiments (Armaniter and Boly 2012; Camerer 2012; Whang 2009; Armantier & Boly, 2008; Abbink and Hennig‐Schimdt 2006^12^), it has still to be kept in mind that the included laboratory experiments were mainly conducted on students’ populations and sometimes on non‐representative samples. Nevertheless, the meta‐regression model shows no significant difference between the effects of laboratory and field experiments, as well as between laboratory experiments conducted on students or other subjects.

Furthermore, even though we are confident that we found a complete reflection of the available evidence, the included studies mainly concern high and upper‐middle income countries (twenty studies).

### 6.3 Quality of the evidence

None of the included studies is of poor quality. Only one paper (Songchoo & Suriya, 2012) presents low external and internal validity because it does not include clear details on how the sampling and experiment were conducted (see Table 4). The other studies are clear and detailed in the description of their methodology, allowing for replication. The sample sizes are usually large and the results of experiments presented in details.

### 6.4 Limitations and potential biases in the review process

As discussed above, the main limitation of this review is related to the fact that the majority of studies test different interventions at the same time, or only one intervention but under specific conditions. This issue might affect the comparability of the different experiments.

Besides this issue, other biases might be related to the lack of a standard classification of corruption and anti‐corruption interventions. Due to the multifaceted nature of corruption, the types of corruption considered for this review may overlap over specific characteristics. Consequently, the different categories of corruption are not mutual exclusive. Similarly, also the macro categories of interventions are not mutual exclusive. However, in both cases, it was always possible to identify one “dominant” characteristic of corruption or intervention and to attribute it to only one macro category of analysis.

Another potential limitation of this review concerns the date of the search. Indeed, the main literature search has been conducted in 2018. However, considering the scarcity of randomized controlled trials for assessing the impact of anti‐corruption policies, the likelihood that the results of this review are still updated after three years is very high.

### 6.5 Agreements and disagreements with other studies or reviews

There are not similar studies or reviews using the same methodology we used for our review. Therefore, it is not possible to perform this comparison.

## 7 AUTHORS’ CONCLUSIONS

### 7.1 Implications for practice and policy

The results of this systematic review provide policy makers and practitioners with evidence that can be used to orient anti‐corruption policies.

Our results suggest that interventions based on control and deterrence are effective in curbing administrative corruption, with the following nuances: a) increasing the probability of detection reduces corrupt practices, especially through tightened monitoring systems; b) increasing the severity of sanctions reduces offering and taking bribes; c) increasing positive incentives help to promote compliant behavior. These types of interventions are more effective than organizational and cultural reforms (e.g. promoting codes of ethics, increasing the transparency of information, rotating staff, decentralizing public service), at least in the short term and under specific circumstance linked to the characteristic of the participants to the experiment. Indeed, even if the results of the meta‐regression analysis confirm there are no differences between RCTs conducted with students as participants and those with other types of participants, the generalizability of results might be affected by other specific participants’ characteristics that have not been considered in this analysis (e.g., gender, age, level of education). This is especially true when considering that our analysis is based on 25 laboratory experiments.

The combination of different interventions proves to be more effective than single interventions. This is true not only when combining policies reinforcing control and deterrence (e.g., monitoring frequency, detection probability and amount of fines), but also when policies based on organizational and cultural change are added (e.g., staff rotation and leniency).

For example, in the former case, reporting systems and policies guaranteeing impunity to officials or citizens who report corrupt practices (principal witness/leniency treatment) are effective if associated with a high probability of audit, in a simulated business situation with two bidders competing for a contract placement (Christöfl et al., 2017). Low detection probability can effectively reduce both the amount and the likelihood of bribe demand when associated to high fines. However, according to a lab‐experiment conducted with undergraduate students at a large public university, a high probability of detection does not reduce the likelihood of corrupt practices and the number of bribes as long as sanctions are too low (Banerjee & Mitra, 2018). Indeed, Zamboni and Litschig (2018) suggest that the success of audit in preventing corruption is strongly dependent on the seriousness of the potential sanction and the probability that a sanction will be applied.

With regard to the combination of policies based on both control and deterrence and organizational and cultural change, Abbink and Wu (2017) argue that reporting mechanisms and leniency policies (both based on deterrence) increase their potential in combination with interventions that limit agent's exposure to one another – such as staff rotation (based on organizational change). They demonstrated this through a game in the laboratory simulating a reward mechanism.

The results of the abovementioned studies also demonstrate that the impact of the intervention is affected not only by the size and probability of fines, and the size of the bribe, but also by the likelihood of the continued interactions between the client and the agent.

In addition, the results of the included studies reinforce the importance of a moral understanding of corruption that goes beyond a merely economic explanation of this phenomenon. Indeed, Armantier and Boly (2014), through an experiment where graders were offered a bribe to report a better grade, show that the effect of bonuses and penalties might backfire as they lower the moral cost of corruption by diverting subjects away from their sense of duty and their ethical responsibilities. Following the Clashing moral values theories (De Graaf, 2007: 53), if corruption emerges as a clash between the particularistic values of public officials and the values linked to their official role, simply increasing their wages would not lower the importance of their moral personal duties to friends and family (Jancsics, 2019). These duties, and their related social consequence, will still overrule the agents’ obligations as a public officer. The social costs they would face by refusing to “help” their informal social network would, indeed, be extremely serious in terms of social exclusion, and sometimes even lead to physical violence (Jancsics, 2019: 9). These mechanisms are particularly strong when the client (potential corruptor) and the agent (public official) are members of the same social network outside the public organization and therefore subject to the same informal normative systems (Jancsics, 2019: 9).

The importance of moral levers in preventing corruption also emerges in the study of Falisse and Leszczynska (2015) where they demonstrate that sensitization messages stressing public officials’ professional identity, values, and position increase the moral cost of bribery and negatively affect bribe demands among public servants. However, bribe‐taking seemed not to be affected by these moral messages (Falisse & Leszczynska, 2015).

In order to be effective, anti‐corruption policies should be targeted to specific types of corrupt behaviors, and consider also the level of trust between corrupt partners. As discussed by Jancsics (2019), in case of social bribery aimed at fulfilling social obligations (e.g., providing a job to a family member, avoid a fine to a friend, etc.), external top‐down measures (e.g., anti‐corruption regulations, law enforcement authorities, government monitoring of organizational operations) are not just ineffective but may even increase this type of corruption. Indeed, in case of social bribery, informal norms and ethics often override formal rules. However, if the public official and the potential corruptor do not belong to the same informal network and are not affected by the same informal normative system, asymmetric (top‐down) penalties imposed by external authorities and the punishment of the public official only might increase the chance of reporting (Jancsics, 2019).

Another relevant issue in terms of policymaking concerns the importance of distinguishing between high and low corruption countries when evaluating the effectiveness of anti‐corruption interventions. The results of Campos‐Vazquez and Mejia's (2016) laboratory experiment on with university students in Mexico demonstrates that the same public policy applied in different contexts (low and high corruption) may produce very different outcomes.

Timing and endurance of interventions are also important to consider when designing anti‐corruption policies. Benerjee and Mitra's (2018) laboratory experiment demonstrates that ethics education can reduce the likelihood of bribe demand (but not the amount of bribe demanded) only in the short term (not more than four weeks).

### 7.2 Implications for research

The fact that control and deterrence turn out to be more effective than organizational and cultural interventions in curbing administrative corruption confirms the importance of economic theories (and cost benefit analysis) in understanding corruption mechanisms. However, the meta‐analysis also demonstrates the effectiveness of combining interventions of both types. In particular, the impact of moral levers in preventing corruption highlights the need of going beyond economic and principal agent models and considering also the moral and cultural mechanisms for explaining corruption.

Several authors consider the predominance of the economic theories for explaining corruption as the leading cause of the failure of anti‐corruption policies (Marquette & Pfeiffer, 2015; Persson et al., 2013; UNDP, 2015; Johnsøn 2012). Other authors point to the design‐reality gap (Heeks and Mathisen 2011), stemming from the lack of understanding of relevant social contexts, and from a simplistic definition of corruption (Heywood, 2017: 12).

The results of our review also confirm the need for anti‐corruption interventions to be targeted at specific types of corrupt behaviors in order to be effective, and the need of understanding how different forms of corruption operate in practice at macro (cross‐country), meso (country/nation‐state) and micro (individual) level (Heywood, 2017: 9).

At the macro‐level, it would be interesting to investigate the influence of international geo‐political and financial forces on the emergence of new transnational corruption networks. At the nation‐state, or meso‐level‐ the nation‐state‐ we would need to understand the specific characteristics of a country that might influence the effectiveness of anti‐corruption policies (e.g., historical development, institutional configurations, socio‐economic organization and particular corruption issues). What does make specific sectors more at risk of corruption (e.g., public procurement, constructions, etc.) than others? At micro level, the focus should be on “how and why individuals engage in various different kinds of corruption, moving beyond the basic incentives‐based model of instrumental rationality that has underpinned much economic analysis” (Heywood, 2017: 11). In particular, individual‐level factors, such as the strive for power, low self‐control, loss aversion and risk acceptance would need to be addressed (Dupuy & Neset, 2018).

When more experimental studies will be available, it would be interesting to distinguish between top‐down (from supervisors to officials) and bottom‐up (from citizens to officials, i.e., I Paid a Bribe website) monitoring systems. Ryvkin, Serra, and Tremewan (2017) show there could be interesting results in this regard. Indeed, the effectiveness of bottom‐up tools, such as whistle‐blowing, staff morale, or community monitoring, has attracted little attention from empirical researchers (Gans‐Morse et al.; Jancsics, 2019), but it can be particularly effective where moral levers play an important role (e.g., bribery and corruption to “help” relatives and friends in getting a job).

From a methodological point of view, it could be tested whether the effects of anti‐corruption interventions change according to the types of game used in corruption experiments^13^ (e.g., behavioral game theory, trust game, etc.), and according to the setting in which the experiment was conducted (e.g., context‐free versus in‐context presentation of experimental tasks) (see Abbink and Schmidt 2006; Abbink and Henning 2006).

Considering the results of Falisse and Leszczynska (2015) on the effect of sensitization messages in reducing bribery demand, we would encourage researchers to develop other corruption experiments exploring the role of professional self‐identity and family corporate culture on corruption.

Furthermore, the review highlights the need for a comprehensive classification of anti‐corruption policies that distinguishes interventions by types of corruption, risk factors, types of policy tools, and characteristics of public sector.^14^

## Information about this review

### Review authors

#### Lead review author

The lead author is the person who develops and co‐ordinates the review team, discusses and assigns roles for individual members of the review team, liaises with the editorial base and takes responsibility for the on‐going updates of the review.

### Roles and responsibilities

- Content: Giulia Mugellini and Martin Killias
- Systematic review methods: Marco Colagrossi
- Statistical analysis: Marco Colagrossi, Sara Della Bella, Giulia Mugellini, and Martin Killias
- Information retrieval: Sara Della Bella, Marco Colagrossi, and Giulia Mugellini.
- Title registration and first version of the protocol: Martin Killias, Giulia Mugellini, and Giang Ly Isenring

### Sources of support

Financial support for this study has been provided by the University of St. Gallen (HSG).

### Declarations of interest

None of the authors has any conflict of interest in the outcome of the review.

### Plans for updating the review

This review will be updated every three years to include new anti‐corruption reforms. The primary authors will take the lead in future updates.

## Supporting information

Supporting information

## Footnotes

^1^

https://www.oecd.org/corruption/countryreportsontheimplementationoftheoecdanti-briberyconvention.htm.

^2^

https://www.oecd.org/corruption/acn/istanbulactionplancountryreports.htm.

^3^
One year after the completion of this review, Villeneuve et al. (2019) identified one common problem of these existing classifications: the lack of consideration of the existing policy tools. The authors address this issue and suggest a new typology of anti‐corruption interventions that considers four main elements: Type of Policy Tool, Mechanism of prevention/intervention; Motivation for Corruption; Type of Gain.

^4^
Considering too many categories of interventions with a relatively small sample in a context of high‐between heterogeneity of size could have led to either spurious or non‐reliable relationships. Importantly, high‐between heterogeneity is common among social science meta‐analysis, and the levels found in our analysis are in line with levels found in many other meta‐analyses (see Ioannidis et al., 2017).

^5^
In particular, increasing the graders' wages reduces in both environments the probability to accept a bribe (Armantier and Boly, 2008).

^6^

http://datatopics.worldbank.org/world-development-indicators/the-world-by-income-and-region.html.

^7^
The analyses presented in this review have conducted using the R software package Metafor (Viechtbauer, 2010).

^8^
The analysis have also been conducted using raw partial correlation coefficients and no sizable differences have been detected.

^9^
This situation refers to studies focusing on political corruption or private‐to‐private corruption, for example, instead of administrative corruption. Or when corruption is considered as theft. In Belot (2013) corruption was expressed as theft from students who volunteer to do a certain job. In Murray (2017) corruption was proxied by the emergence of alliances between players. We did not evaluate back‐scratching as an eligible type form of corruption for our review. The study of Cheng (2009) refers to private‐to‐private corruption.

^10^
This situation refers to studies measuring the outcome not as an experience of corruption but as a perception of corruption, or willingness to corrupt someone. In Samia Tavares (2007) corruption was measured by ICRG index (International Country Risk Guide) and ICP (corruption perception index). Corbacho et al. (2016) evaluate the impact of information on the willing to bribe a police officer and not on corruption experiences.

^11^
Importantly, results are not only robust to different models, but also to different heterogeneity estimators. In fact, while in this review the parameter $\tau^{2}$ is fitted using the restricted maximum likelihood (REML) estimator, other estimators, namely the maximum likelihood (ML), the De‐Simonian‐Laird (DL) and the Hedges estimator (HE) provide highly comparable results.

^12^
Abbink and Hennig‐Schimdt (2006) hypothesized that loaded instructions during laboratory experiments reduce the level of corruption (or equivalently, that neutral instructions take away the negative connotations of it and thus increase the level of harmful reciprocation). However, they their results “exhibit only mild effects of the treatment, and we do not find significant differences. This is surprising as our manipulation appears far stronger than the ones reported in the literature mentioned earlier. We conjecture that the experimental design transmits the essential features of a bribery situation already with the neutral framing, such that the presentation does not add substantially to subjects’ interpretation of the task (Abbink and Hennig‐Schimdt 2006: 105).

^13^
“The typical corruption game consists of a bribery game between two individuals, the proponent and the respondent. Proponents have to decide whether to offer a bribe to respondents and if so, the amount to be offered. Respondents then decide whether to accept the bribe, and at the same time the value of the favour to be paid” (Campos‐Vazquez and Mejia 2016: 3).

^14^
See subsequent paper of Villeneuve et al. 2019.

## APPENDIX 1 – SUMMARY OF INCLUDED STUDIES

The studies are grouped by type of intervention (starting from control and deterrence interventions and then addressing organizational and cultural interventions) and listed in ascending order by year of publication.

### Monitoring interventions

1. Olken
  ^15^
  Olken, B. A. (2007). Monitoring corruption: evidence from a field experiment in Indonesia. Journal of political Economy, 115(2), 200‐249.
   (2007) developed a field experiment during which he found out that top‐down monitoring worked better than informal monitoring in reducing corruption in 608 Indonesian village road projects. The author wanted to see whether increasing monitoring and different type of monitoring (top‐down or bottom‐up/grassroots) could reduce corruption measured as percent missing expenditure for roads (i.e. discrepancies between official project costs and an independent engineers’ estimate of costs). More precisely, in his field experiment, Olken investigated the anti‐corruption effect of three interventions: 1) increasing the probability of external audits from government (from 4% to 100%), 2) increasing community members’ participation in accountability meetings through invitations, 3) increasing community members’ participation in accountability meetings through invitations, plus the provision of an anonymous comment form to villages. The idea behind the grassroots approach is that community members are ones who benefit from a successful program and so the ones who may have better incentives to monitor, though there might be problems such as that of free rider and of capturing of grassroots monitoring by local elites. Indeed, the author found out that traditional top‐down monitoring was able to reduce corruption (missing expenditures decreased by 8 percentages points), at least in the short run, whereas increasing grassroots monitoring seemed to have a limited impact on corruption. Grassroots monitoring worked only in reducing missing labor expenditures, but had no impact on materials and, as a consequence, little impact overall. Moreover, issuing anonymous comment forms to villagers reduced missing expenditures only if the comment forms were distributed via schools, completely bypassing village officials who may have been involved in the project. These results suggest that grassroots monitoring could work only under special circumstances: little free riding and limited elite capture.
2. **Serra (2012**)
  ^16^
  Serra, D. (2011). Combining top‐down and bottom‐up accountability: evidence from a bribery experiment. The journal of law, economics, & organization, 28(3), 569‐587.
   investigated the effect of monitoring on corruption through a laboratory experiment. He studied the effect of both endogenous and exogenous monitoring on collusive corruption. In this case, subjects were randomly allocated the roles of public official, private citizen, and other member of society. The public official had the possibility of demanding a bribe from a private citizen in exchange for the provision of a better or quicker service. The author then conducted three different versions (i.e., treatment) of the game to compare public officials’ tendency to ask for bribes in one shot interactions under: 1) no monitoring (corrupt officials immune from formal fines); 2) exogenous monitoring (i.e., a conventional top‐down auditing where detection happens with a certain probability), 3) a combined accountability system whereby citizens had the possibility of anonymously reporting corrupt officials, knowing that reports lead to top‐down auditing with some low probability (the same as in the second treatment: 4%). The experimental results suggest that in the last treatment less and lower bribes are asked. More precisely, the combined monitoring system had the only significant effect on the size of the bribe demanded and it reduced the probability of a public official demanding a bribe by about 30%, whereas the presence of only top‐down auditing did not affect bribe‐demanding behavior. Hence, combined monitoring seemed to be highly effective in curbing corruption even in a weak institutional environment where both the probability of formal punishment and the fine are low.
3. The study by Khachatryan et al. (2015) tested whether the introduction of a bottom‐up approach (i.e., endogenous monitoring by citizens) could reduce extortive petty corruption. In the laboratory experiment run in August 2012, the recruited subjects were either in the role of the public official (PO) or the citizen. Depending on the official's behavior, citizens could then report a bribe‐asking official or recommend a law‐abiding official. An official who was reported (recommended) then had to pay a fine (could receive a bonus) with a certain probability. Within treatment, this probability varied (it could be 5%, 10% or 20%). The authors tested the effect of both endogenous monitoring mechanisms in different setups: in personal and repeated interactions (partner matching) and in one shot interactions (stranger matching). Results were mixed. In the stranger matching, public officials were more likely to ask for a bribe. Otherwise, citizens were less likely to pay the demanded bribe in case of partner matching. Independent of matching protocol and monitoring mechanism, public officials were sensitive to monitoring and detection rate variations in their decision to demand a bribe. Indeed, higher rates of being fined (or rewarded) lead to less bribes being demanded. In particular, this holds in the stranger treatments, whereas in the partner treatments public officials seem to be more likely to continue asking for bribes.
4. Campos‐Vazquez and Mejia (2016)
  ^17^
  Campos‐Vazquez, R. M., & Mejia, L. A. (2016). Does corruption affect cooperation? A laboratory experiment. Latin American Economic Review, 25(1), 5.
   conducted a laboratory experiment in Mexico to test the effect of high and low monitoring scenarios on corruption. They replicated a situation where there is a firm (or citizen) that wishes to install a factory next to a river and needs an environmental certificate from the local government. The firm has the possibility of offering the official a bribe as an incentive to grant the certificate. In the high‐monitoring scenario the firm (which is the one offering the bribe) and the official (the one that has to decide whether to accept it or not) face a 25% probability of being caught. In the low‐monitoring scenario the probability of being caught is of 5%. The bribe is not demanded by an official and paid by a firm, but offered by the firm in order to get a special permit, and accepted by an official. The results showed that individuals in the high‐monitoring group commit fewer corrupt acts than those in the low‐monitoring groups (52% offered a bribe against 35%). This is true both for individuals acting as firms and for those acting as public officials. Second, the high‐monitoring group offers a larger bribe than the low‐monitoring group, possibly with the intent of increasing the probability of receiving the certificate given the higher risk of getting caught. Third, individuals with greater temptation to corruption (a lower probability of getting caught) cooperate less with each other than individuals with lesser temptation (a higher probability of getting caught). Fourth, adding the option of punishment increases cooperation. This increase is stronger in low‐corruption scenarios.
5. The study of Salmon and Serra (2017)
  ^18^
  Salmon, T. C., & Serra, D. (2017). Corruption, social judgment and culture: An experiment. Journal of Economic Behavior & Organization, 142, 64‐78.
   investigated the effect of social observability and the possibility of social judgment (hence a sort of informal monitoring, involving informal sanctions) on individual's decisions to engage in corruption at the expense of others. In the laboratory experiment, a subject in the role of citizen chose whether to offer a bribe. If the public official decided to accept and the transaction happened, then a third member of society suffered a monetary loss. The authors manipulated the degree of social observability of citizens and social sanctions so that the game was played under three different circumstances. In the first treatment, citizens and officials knew that their corrupt actions will be hidden from others, in the second they knew that their actions will be visible to the victim and in the third they knew that others, including the victim, would judge their behavior. The authors also wanted to understand whether the effect of social judgment varies with culture (i.e., according to the country with which subjects identify, classified as a low or high corruption country), while holding institutional environment and monetary incentives constant. Hence they repeated the analysis separately for subjects identifying with low corruption country and for subjects identifying with high income country. Overall, Salmon and Serra found out that corruption weakly decreases as social observability increases and is lowest when society is given the chance to express social approval or disapproval against corrupt individuals. However, culture matters in that the possibility of social judgment reduces corruption only among individuals who identify culturally with countries characterized by low levels of corruption.
6. Zamboni and Litschig (2018) conducted a randomized policy experiment to test whether increased audit risk deters rent extraction in three areas of local government activity in Brazil: procurement, health service delivery and cash transfer targeting. The annual audit risk was increased from a baseline level of about 5 percent to about 25 percent across the experimental group of local government activities. Their results suggest that “increasing annual audit risk by about 20 percentage points reduced the share of audited resources involved in corruption in procurement by about 10 percentage points and the proportion of procurement processes with evidence of corruption by about 15 percentage points” (Zamboni & Litschig, 2018: 133). They also found that their results are invariant to alternative corruption definitions. In contrast, they found that the increased audit risk did not affect the quality of publicly provided preventive and primary health care services or compliance with eligibility requirements for the conditional cash transfer program. The authors argued that the different impacts of audit risk in procurement vs. health service delivery is mainly due to the seriousness of the potential punishment/sanctions and the probability that a sanction is applied. Indeed, “for service delivery irregularities, sanctions include at most the loss of the job. For public officials in charge of procurement in contrast, potential sanctions are relatively high as they include not only job termination but also fines as well as jail time” (Zamboni & Litschig, 2018: 135). Similarly, “The sanctioning probability in service delivery is likely low because irregularities in service provision cannot be unambiguously identified through an audit. … In contrast, irregularities in procurement are relatively easy to prove because local officials are required to document each step of the purchasing process” (Zamboni & Litschig, 2018: 135).

### Public officials’ wages

7. Azfar and Nelson (2007)
  ^19^
  Azfar, O., & Nelson, W. R. (2007). Transparency, wages, and the separation of powers: An experimental analysis of corruption. Public Choice, 130(3‐4), 471‐493.
   are among the first who studied the effect of wages (among other variables) on corruption. Following Klitgaard's (1988) definition of the institutional conditions under which corruption exist (i.e. Discretion + Monopoly – Accountability), they focused on the role of accountability in mitigating the executive's corruption, defined as the abuse of office for personal gain. Accountability consists of the costs and probabilities of being caught. Hence, in order to study its effects, the authors varied experimentally the cost of being caught (i.e. the wages) and the probability of being caught (i.e. transparency and separation of power). The experiment confirmed theoretical predictions according to which higher government wages and the difficulty of hiding corrupt gains reduced the level of corruptions (defined as the number of valuable tiles the executive keeps for himself). However, the data did not confirm the hypothesis that the direct election of the attorney general should be negatively related to the level of corruption.
8. Armantier and Boly (2008) run the same experiment in a laboratory in Canada as well as in the field in Burkina Faso and found out that, after controlling for individual characteristics, the treatment effects they measured were statistically indistinguishable between the lab and the field. Compared to the study of Van Veldhuizen (2013) (see below), Armantier and Boly investigated the effects of wages on corruption in one‐shot games rather than in long term relationships. More precisely, participants in the lab or field experiments had to grade homework and, in one of the homework sets, they received a bribe accompanied by a request to be lenient in grading. The authors manipulated the wage of graders (increased by 40% compared to the control treatment), the amount of the offered bribe (doubled compared to the control treatment) and the presence of a monitoring mechanism (i.e., the introduction of a monetary penalty based on the worst graded exam) to see whether any of this factor affected the grader's probability of accepting the bribe. The authors found out that increasing graders’ wages significantly lowered their probability of accepting the bribe (after controlling for gender, age, religiosity, ability at grading and time used to complete the task) in both the lab and the field. They also found that increasing the amount of bribe had no effect in the lab, whereas it increased corruption in the field. Monitoring did not affect the decision to accept a bribe in any of the two environments.
9. In a later paper, Armantier and Boly (2014)
  ^20^
  Armantier, O., & Boly, A. (2014). On the effects of incentive framing on bribery: evidence from an experiment in Burkina Faso. Economics of Governance, 15(1), 1‐15.
   conducted a similar experiment in Burkina Faso, in which graders are offered a bribe to report a better grade, in order to test whether incentive framing, that has been shown to improve workers’ effort, had also an impact on corrupt behavior. More precisely, the authors investigated whether the graders’ propensity to accept the bribe varied according to the framing of economically equivalent contracts as menus of bonuses, penalties, or bonuses and penalties. The study showed surprising results: no significant difference in attitudes toward corruption appeared when employment contracts are framed as a menu of bonuses or as a menu of penalties. This is in contrast with the “labor fairness and reciprocity theory”, according to which if the worker feels he is treated fairly, then he/she is more likely to reciprocate by taking actions favorable to the employer and, hence, workers who are offered contracts framed with higher bonuses are supposed to refrain from engaging in corruption. Further, in contrast with standard economic theory predicting that agents respond only to monetary payoffs and hence that the graders’ behavior should be the same in all three treatments, the study found that graders facing a combination of bonuses and penalties are more likely to accept the bribe (+ 21%) and report fewer mistakes for the bribe paper. According to the authors, a possible explanation is that “by crowding out intrinsic motivations, incentives framed as bonuses and penalties divert subjects away from their sense of duty and their ethical responsibilities, thereby lowering their moral cost of corruption”.
10. Barr et al. (2009)
  ^21^
  Barr, A., Lindelow, M., & Serneels, P. (2009). Corruption in public service delivery: An experimental analysis. Journal of Economic Behavior & Organization, 72(1), 225‐239.
   enrolled Ethiopian nursing students to investigate the effects of the institutional environment on the behavior of health service providers and their monitors. Evidence suggested that in Ethiopia health workers often expropriate consumable resources for use in private or semi‐privatize the public clinics by charging so called “side payments” for services rendered. The authors hence investigated possible factors associated with corruption conducting a Public Servant's Game involving eight players, who in different rounds may play the role of “community member,” “public service provider” and “monitor.” In their experiment, the authors varied four different factors: whether the monitor was randomly selected or elected by the community; monitor's observability (high vs low); the wage of the service provider (high vs low) and the framing of the game as a public health provision scenario (i.e. as a context in which professional identities are relevant). As it concerns the factors associated with misappropriation by service providers, results showed that service providers performed better when monitors are elected by service recipients and when their effort is more easily observed. Contrary to expectations, the wage of service providers was only weakly associated with their performance. Framing the study as a public health provision scenario was associated with higher level of corruption in service providers, suggesting that experience and norms affect behavior.
11. Songchoo and Suriya (2012)
  ^22^
  Songchoo, T., & Suriya, K. (2012). Competition to commit crime: An economic experiment on illegal logging using behavioural game theory. The Empirical Econometrics and Quantitative Economics Letters, 1(1), 75‐90.
   developed an economic experiment using behavioral game theory to investigate what intervention might have a significant negative effect on illegal logging in Thailand. In particular, they tested the effect of two main interventions on bribery regarding police officers: high punishment with low reward and low punishment with high rewards. Their results showed that the high reward policy was effective to help reducing the illegal logging problem.
12. In a laboratory experiment conducted between 2010 and 2011, Van Veldhuizen (2013)
  ^23^
  Van Veldhuizen, R. (2013). The influence of wages on public officials’ corruptibility: A laboratory investigation. Journal of economic psychology, 39, 341‐356.
   examined whether increasing public officials’ wage reduce their likelihood of accepting a bribe and deciding for a corrupt action that would benefit the briber, but would pose a large negative externality on a charity. The wage effect was tested in a repeated game of 25 periods, that mimicked the creation of a long‐term relationship between the citizen and the public official, as this kind of relationship is particular likely to develop in petty corruption scenario. Van Veldhuizen choses as the reference wage the briber's one and, in the experiment, he manipulated the public official's wage so that it was either equal to the income of the briber or higher. The author also included a monitoring mechanism: If the public official accepted the offer, there was a small probability (.003) that both players would be caught and received a punishment. Results of this study show that increasing public officials’ wages greatly reduces their corruptibility. More precisely, the wage increase made public officials 53 percentage points less likely to accept a bribe and reduced the number of corrupt choices by 27 percentage points. Additionally, results of a robustness check suggested that the wage effect might be conditional on a non‐zero level of monitoring.
13. Banerjee and Mitra (2018)
  ^24^
  Banerjee, R., & Mitra, A. (2018). On monetary and non‐monetary interventions to combat corruption. Journal of Economic Behavior & Organization, 149, 332‐355.
   wanted to identify the relative strengths and weaknesses of extrinsic monetary disincentives and intrinsic non‐monetary disincentives to corruption using an harassment bribery game. The authors actually conducted two experiments on different subject pools. Both experiments were framed as a situation in which a citizen is entitled to a prize and the public official can decide whether to approve it and ask for a bribe, but treatments changed between the first and the second experiments. In the first, the authors compared the effects of two different combinations of monitoring and sanctions: low probability of audit for public official with high fine; and high probability of audit with low fine. The second experiment focused on the moral cost or intrinsic disincentive for demanding bribe and tested the efficacy of an ethics teaching module by varying whether a subject goes through the ethics module or not and when the outcome of interest (demanding bribe) is observed (1 week or 4 weeks after the module). In this way the authors could test whether ethics education's effects, if any, hold longitudinally. Results from the first experiment show that low probability of detection and high fine significantly reduced both the amount and the likelihood of bribe demand, but high probability of detection and low fine had no effect on either. Results from the second experiment show that ethics education did not have any effect on the amount of bribe demand neither in the short nor in the long term. However, it did have an effect on the likelihood of bribe demand in the short term, but not in the longer term (i.e., ethics education effect does not survive beyond the fourth week).

### Reporting Corruption

14. Ryvkin, Serra, and Tremewan (2017) investigated the impact of online reporting platform where citizens can post their experiences of bribes demand by pubic officials (extortionary corruption). The authors took as baseline setting of the reporting platform the *I paid the bribe* website, and they add the possibility to search for the least corrupt officials. In this way, they could investigate the impact that an online reporting platform would have on bribe demands if it could also be used as a search engine for low‐bribe‐demanding officials. The authors developed a laboratory experiment and designed a treatment where citizens can leave posts not only about the size of the bribe demanded but also about the specific location of the office/official that demanded the bribe. In addition, they created a third version of the platform where were both citizens and official can post (BB_ALL). In their experimental setting citizens need to obtain a license and public officials can ask for bribes on top of the official licensing fee. Citizens can obtain the license from any of the available offices displayed on a map; however, every time they visit a new office they incur a cost. They have three interventions based on three different website/maps: *I paid a bribe* (BB_IPB) is a website where they can report the bribe demands, enhanced version of I paid a bribe where also the location of the office/official were the bribe has been demanded has to be reported (BB_CIT), version were both citizens and official can post (BB_ALL). Their results showed that the presence of a reporting platform like the *I paid a bribe* website may be insufficient to systematically lower bribery. However, if the website could contain information not only on the size of the bribes but also on the location of the offices where such bribes where demanded, harassment bribes would be significantly lower. Restricting the use of reporting systems to service recipients further reduces corruption. This is because when officials are allowed to leave messages on the reporting platforms, they tend to post multiple false messages, compromising in this way the credibility and efficacy of the reporting system. On the other hand, they notice very little lying from citizens.

### Leniency

15. Abbink et al. (2014)
  ^25^
  Abbink, K., Dasgupta, U., Gangadharan, L., & Jain, T. (2014). Letting the briber go free: an experiment on mitigating harassment bribes. Journal of Public Economics, 111, 17‐28.
   investigated the role of asymmetric liability against bribery, whereby bribe‐takers are culpable but bribe‐givers have legal immunity. This approach could potentially reduce harassment bribes by encouraging more frequent reporting from citizens and, hence by discouraging officials from demanding bribes in anticipation of the whistleblowing. To examine the effectiveness of the asymmetric liability mechanism in combating harassment bribes, the authors conducted a laboratory experiment in which they varied the institutional environment in order to identify conditions under which this kind of policy measures may work. More precisely, the experiment consisted in a simple sequential‐decision game between an official and a citizen for the delivery of a service that the latter was entitled to. Since the official had de facto the discretion to deny or delay it indefinitely, he could demand a bribe for speedy delivery and the citizen could accept or refuse to pay (with some costs). The authors hence examined whether and how official's and citizen's choices varied under symmetric and asymmetric treatments. Moreover, they added two additional treatments by a) introducing the possibility of retaliation by officials in the asymmetric liability scenario and b) by manipulating the practicality of bribe‐returns to the citizen (while leaving the retaliation option). The percentage of officials demanding a bribe dropped from 38% to 24% when moving from symmetric to asymmetric liability. However, the proportion of officials demanding bribes increased again in the retaliation and no refund treatments (38 and 27% respectively). Furthermore, in these two additional treatments the amount of bribe demanded were lower than in the symmetric treatment but higher than in the pure asymmetric treatment. Overall, this study provides support for asymmetric liability as an anti‐corruption mechanism by showing that, when bribe‐giving was legalized, reporting increased and demands for bribes decreased. This effects persisted even when bribes were not refunded after prosecution, showing that intrinsic motivation was the main driver of citizens' reporting behavior. However, this study also showed that retaliation can significantly dampen the effects of the asymmetric treatment. Hence, asymmetric liability should be implemented along with complementary measures to protect citizens from retaliation
16. Bigoni et al., 2015
  ^26^
  Bigoni, M., Fridolfsson, S. O., Le Coq, C., & Spagnolo, G. (2015). Trust, leniency, and deterrence. The Journal of Law, Economics, and Organization, 31(4), 663‐689.
   developed a laboratory experiment studying law enforcement strategies to deter cartel formation. They tested the effect of four main interventions: fine sizes and probability of detection by a law enforcement agency, possibility of reporting and reporting effect on collusive corruption. In particular, they tested the following combinations of interventions: FINE, fine goes from 200 to 1000, probability to get caught from 0% to 10%, it is possible to report, but if ones report it will pay the full fine. LENIENCY: fine goes from 200 to 1000, probability to get caught from 0% to 10%, it is possible to report, but if ones report, it won't pay the fine. L‐FAIRE, no fine, no probability of being caught and no possibility to report. NOREPORT has fine 200 and detection probability of 10%. Their results suggest that leniency policies restricted to the first party to report spontaneously, without being subject to an investigation, significantly decrease collusive corruption. This decrease appears to be mainly driven by the fear of being betrayed and reported. By increasing both the incentive to betray and the cost of being betrayed by a partner, leniency seems to generate a higher demand for trust among criminals, hence less crime for any given level of trust. Then, a high absolute level of the fine is the most important determinant of deterrence, effective even if there is no risk of detection by the law enforcement agency without a report” (Bigoni et al., 2015: 19).
17. Engel et al. (2016)
  ^27^
  Engel, C., Goerg, S. J., & Yu, G. (2016). Symmetric vs. asymmetric punishment regimes for collusive bribery. American Law and Economics Review, 18(2), 506‐556.
   also investigated the possible effects of different punishment regimes on collusive bribes. These punishment regimes are the two prevalent ones in major legal orders with either symmetric punishment of briber and receiver or asymmetric punishment favoring the briber. An asymmetric approach has been showed to decrease harassment bribes (Abbink et al., 2014), however, Engel et al (2014) argued that asymmetry might result in reinforcing collusive bribes. The authors tested for the occurrence of this potential downside by running the same lab experiments in Bonn (Germany) and in Shanghai (China) in order to see whether results reflect a generalizable effect, rather than differences in national cultures, or in the legal environment. In their bribery game, a citizen can offer a bribe to an official, who can reciprocate by manipulating his decision in the citizen's favor. In the first set of experiments, the authors adopted a neutral frame and found out that under asymmetric punishment bribers are more likely to report to the authorities if the recipient accepts the bribe but does not grant the favor. On their side, recipients are slightly less likely to accept the bribe, but if they do, they are much more likely to grant the favor. In the second set of experiments, the authors implement a loaded frame (calling the transaction a bribe and so on) to understand whether potential moral compunctions change the results. Overall, it appeared that punishing the briber in collusive bribery more leniently is a very bad idea. Indeed, in both countries, symmetric punishment seemed to reduce the propensity of bribers to report to the authorities and of officials to grant the favor. In Shanghai, symmetry also reduced corrupt offers. However, these results are not necessarily transferrable to harassment bribes.
18. Abbink and Wu (2017)
  ^28^
  Abbink, K., & Wu, K. (2017). Reward self‐reporting to deter corruption: An experiment on mitigating collusive bribery. Journal of Economic Behavior & Organization, 133, 256‐272.
   investigate, through a laboratory experiment of a bribery game, the effectiveness of offering rewards for agents who self‐reports corrupt actions in combating collusive corruption. Several studies in anti‐cartel formation have suggested that offering rewards (sometimes referred to as “bonus” leniency, see Aubert et al., 2006; Jacquemet and Rullière, 2006) may have several benefits over partial or full leniency (Abbink & Wu, 2017: 257). The authors tested three variations of the reward mechanism: both client and official may self‐report (symmetric reporting mechanism), only the client may self‐report and only the official may self‐report (asymmetric reporting). They found that symmetric reporting is the only one effective mechanism in reducing the size of bribes offered and the incidence of bribes being exchanged. Permitting only one party from self‐reporting does not significantly deter bribe exchange. The efficacy of reporting mechanisms was most pronounced in the uncertain continuation periods. This suggests that the potential for reporting mechanisms to deter corruption may be realized only in conjunction with other policies that limit agents’ exposure to one another such as staff rotation. The reward mechanisms did not increase the likelihood an agent would betray their partner except when the agent was themselves exposed to the risk of incurring a fine. Their results support the implementation of a symmetric reward mechanism to deter collusive bribery but they are unable to say whether this mechanism would be effective in combating larger scale bribery or cases where the clients interact with a difference official each time.
19. Christöfl et al. (2017)
  ^29^
  Christofl, A., Leopold‐Wildburger, U., & Rasmußen, A. (2017). An experimental study on bribes, detection probability and principal witness policy. Journal of Business Economics, 87(8), 1067‐1081.
   conducted a laboratory bribery experiment simulating negotiations between an employee and two bidders competing for a contract in order to test whether different probability of a bribe being discovered influence the honesty of a contract placing during public bid. They also investigate the effect of cooperating with the authority (principal witness) in combination with a leniency policy in the form of a reduced fine for the all players who cooperate with the authorities. They implemented an audit process that detects a bribe with a high (40%) and low (10%) probability. In addition, they vary whether bidders and employees who chose to engage in a corrupt transaction both have the opportunity to cooperate with the authorities in a broad form of principal witness mechanism for a reduced fine (representing a leniency policy) once an audit takes place. They found out that principal witness deters the offering of bribes but does not reduce the amount of the bribes that are offered. They also demonstrated that especially in the principal witness treatments bidders offering bribes earn significantly more than their honest counterparts. A broad leniency policy thus seems to create an environment that deters the offering of bribes, but at the same time makes them more profitable for bidders if they offer them. They concluded that the principal witness mechanism with an accompanying leniency policy is successful in generating an environment with fewer bribes present, but has the adverse side effect of making bribes especially profitable for bidders.
20. Buckenmaier et al. (2018)
  ^30^
  Buckenmaier, J., Dimant, E., & Mittone, L. (2018). Effects of institutional history and leniency on collusive corruption and tax evasion. Journal of Economic Behavior & Organization.
   focus on the effectiveness of providing legal immunity to the bribe‐giver for whistleblowing as a means to deter collusive bribery, to which they refer to as leniency. Leniency programs, aimed to fight crime through encouraging whistle‐blowing. Following existing literature, (i.e., Christöfl et al., 2017; Spagnolo, 2006), the authors define leniency as the act of forgiveness of observed transgression on the condition that the taxpayer blows the whistle on the corrupted tax official. They developed a controlled laboratory experiment where “they consider a mechanism that offers taxpayers a “safe way out” by blowing the whistle on a corrupted public official and cooperating with auditors. This mechanism resembles a leniency program for tax evasion in which audited tax fraudsters can become whistleblowers” (Buckenmaier et al., 2018: 3). Their results showed that leniency is effective in deterring tax officers from engaging in bribery in the presence of a leniency mechanism. Collusion between taxpayers and tax officers is less frequent because the willingness of tax officers to accept bribes decreases. Furthermore, there is no significant increase in the frequency of bribes being offered.

### Crackdown

21. Banuri and Eckel (2015)
  ^31^
  Banuri, S., & Eckel, C. (2015). Cracking down on bribery. Social Choice and Welfare, 45(3), 579‐600.
   used lab experiments to investigate whether corruption crackdowns inhibit or exacerbate bribery in the long run. Crackdowns (i.e., punishment institutions) may reduce corruption in the long run, if the crackdown signals a new norm, which is then internalized by the corrupt agents. Their main research questions were: Once a crackdown regime is removed, does it have a lasting impact on behavior? Furthermore, does the crackdown have different post‐crackdown effects in societies with weak corruption norms versus societies with strong corruption norms? In order to answer these questions, the authors developed a lab experiment with subjects from two countries ‐ the US and Pakistan – which show differences in overall perceptions of corruption and experience with bribery. Their results show that crackdowns have some impact on bribery behavior in the short term, particularly in low corruption settings (while enforcement is active), but bribery returns to pre‐crackdown levels during the post‐crackdown phase. These results favor the economists’ view that corruption is determined by extrinsic factors. Short run institutions are largely ineffective in altering behavior in the long run in both high and low corruption settings. In particular, sustained legal enforcement may be necessary to constrain corruption, even in societies with weak norms.

### Transparency (Information and Participation) Interventions

22. Di Falco et al. (2016)
  ^32^
  Di Falco, S., Magdalou, B., Masclet, D., Villeval, M. C., & Willinger, M. (2016). Can transparency of information reduce embezzlement? Experimental Evidence from Tanzania (No. 1618). Groupe d'Analyse et de Théorie Economique Lyon St‐Étienne (GATE Lyon St‐Étienne), Université de Lyon.
   investigated experimentally whether increasing transparency and reducing the number of intermediaries in transfer chains can reduce embezzlement. Using a sequential dictator game and a 2×2 factorial design in which they manipulated both the number of intermediaries (1 or 2 compared to no intermediary) and transparency, the authors tested whether the impact of transparency on embezzlement depends on the length of the transfer chain. The dictator is a well‐endowed sender who can make a transfer to a poorly endowed recipient. In the baseline, the transfer can be carried out directly, in the other treatments the number of intermediaries varies as well as transparency of information (i.e. the recipient's knowledge of the amount of the donation). The authors hypothesized that transparency can lower embezzlement by increasing the intermediaries’ moral cost of stealing by making embezzlement visible to the recipient. However, this direct effect of transparency can be seen only on intermediaries in short chains and on the second intermediaries in long chains. In contrast, the first intermediaries embezzle slightly more under transparency, maybe because of the expectation of less embezzlement further down in the chain. Hence, introducing more transparency does not necessarily reduce embezzlement, at least in the absence of sanctioning institutions.
23. In the context of the India's Mahatma Gandhi National Rural Employment Guarantee Scheme (MGNREGS) which is supposed to provide employment at a given wage to all those who request work and use that labor to improve local infrastructure – Banerjee et al. (2016) implemented a randomized experiment to increase accountability and transparency and reduce the number of intermediaries by using e‐governance. The aim of the reform was to reduce fund leakage. According to authors’ definition, this could occur in two ways: “by reporting “ghost” workers on the database and siphoning off the associated payment (people who are reported to be paid but are non‐existent, or exist but have never worked) or by reporting “ghost” days (additional days of reported work by people who actually worked under the scheme but for fewer days than what is reported)” (Banerjee et al., 2016: 23). In the status quo system, funds flowed through four tiers of administrative hierarchy on their way from the Department of Rural Development to the village authority (Gram Panchayat ‐ GP). In the reformed system, fund disbursement to the GP no longer required approval from block or district officials for the submission of the fund request, but GP could simply enter the names of those employed and wages owed in a central database, which automatically triggered fund release into the GP account. The prerequisite for the reform was IT infrastructure to enable GP to connect to the central database. This system allowed to reduce “ghost” workers by easily auditing and verifying that a particular person exists and has been employed. In order to check for any leakage, the authors followed Olken's (2007) method of tracking expenditure, by comparing official records of funds release with actual receipt by beneficiaries. The authors showed that the applied reform not only reduced involvement of higher administrative tiers, but also enhanced transparency and significantly reduced missing expenditures and workdays. The fact that each fund request required a list of beneficiaries enabled a more effective audit process – it eliminated the several‐month lag between fund transfer and wage payment and when the names of those purportedly paid were available to the auditor.
24. Björkman Nyqvist et al. (2017) tested the longer run impact of a local accountability intervention in primary health care provision in Uganda, and in particular on issues such as absenteeism. In particular, two interventions were developed in 2005 and evaluated in 2007 and 2008: “*participation”* and “*participation & information*”. The *participation* intervention involved three types of meetings facilitated by staff from local community‐based organizations. The main objective of these meetings was to encourage community members and health facility staff to develop a shared view of how to improve service delivery and monitor health provision in the community. The *information & participation* intervention also entailed meetings between community members and the health facility employees. In this case, before the meetings took place, the participants were provided with easily accessible quantitative data on the performance of the health provider. These data were collected from facility and household surveys implemented prior to the intervention. Sharing this data allowed to address informational asymmetries between the providers and beneficiaries and to guide the discussion towards issues that potentially could be dealt with locally. The authors found that the intervention focused only on increasing participation, but not reducing possible informational asymmetries, had little impact, while the *information & participation* intervention resulted in “a more engaged community and in large and long‐run improvements in both health service provision and health outcomes” (Björkman Nyqvist et al., 2017: 36).
25. Khadjavi et al. (2017)
  ^33^
  Khadjavi, M., Lange, A., & Nicklisch, A. (2017). How transparency may corrupt− experimental evidence from asymmetric public goods games. Journal of Economic Behavior & Organization, 142, 468‐481.
   investigated the impact of transparency, looking at how its effects embezzlement and cooperation in the provision of public goods depend on punishment. In order to study the interplay of transparency and punishment in countering corruption, the authors conducted a laboratory experiment between 2011 and 2012 to understand to which extent transparency alone may prevent embezzlement; how it interacts with punishment options and, finally, how these channels affect cooperativeness within groups when embezzlement options exist for some members. In repeated linear public goods games‐where only the official could embezzle money unilaterally from the public good, whereas citizens could only contribute to it‐ the authors manipulated the degree of transparency (none, low, when the official is identifiable to citizen, or high when both individual decisions and the identity of the official are common knowledge) and the availability of peer punishment. The study highlighted the complementary nature of transparency and punishment by showing that higher degrees of transparency significantly improve the provision of public goods only if punishment options exist. Indeed, high transparency alone significantly increased the embezzlement of officials relative to situations where individual decisions were not transparent. In contrast, low and high transparency in combination with punishment improved cooperation through two different channels. Punishment combined with low transparency reduced embezzlement of officials relative to the treatments without punishment through stigmatization of the official. In the case of punishment with high transparency, both the official and citizens are made accountable through being punished based on their decisions rather than their type and, hence, cooperation is significantly greater in this treatment.
26. **Makowsky and Siyu** (2018)
  ^34^
  Makowsky, M. D., & Wang, S. (2018). Embezzlement, whistleblowing, and organizational architecture: An experimental investigation. Journal of Economic Behavior & Organization, 147, 58‐75.
   argued that the patterns by which capital and information flow through agents, the sequence of decision‐making, and the opportunities to extract resources or report the observation of missing resources vary with organizational structure. Hence, they investigated whether and how the organizational architectures could reduce embezzlement. The did so by conducting a common pool resource and ultimatum game in which agents are activated sequentially within an organizational architecture wherein they can take a share of the available resources or choose to "blow the whistle", setting all payoffs to zero. More precisely, the authors considered six basic organizational architectures, varying in terms of number of levels and shape: pure horizontal (that serve as baseline comparator for all other structures), two‐level pyramid and inverted pyramid, three‐level pyramid and inverted pyramid and pure vertical. Results suggested that the rates of embezzlement (and whistleblowing) increase with the number of organizational levels. Moreover, horizontal and pyramid structures were more effective at reducing embezzlement compared to inverted pyramid structures. Indeed, the presence of a first‐mover serving as a “leader by example” corresponded with lower net rates of embezzlement within the group than when the structure is inverted such that a last‐mover serves as a “supervisor”. The authors concluded stressing that organizational shape is a potentially attractive policy lever to mitigate embezzlement: the implementation of flatter architectures and the strategic placement of leaders in chains of decision‐making are more accessible strategies compared to costlier ones like explicit monitoring systems.

### Competition

27. Ryvkin and Serra (2016)
  ^35^
  Ryvkin, D., & Serra, D. (2016). The Industrial Organization of Corruption: Monopoly, Competition and Collusion.
   conducted a laboratory experiment in order to test whether introducing competition among public officials by allowing citizens to engage in costly search and get the license from any of the available offices can reduce extortionary bribery (the demand of bribes for the provision of services that citizens are entitled to or qualified to obtain). Their extortionary corruption experiment replicated a situation where citizens need to obtain licenses from public officials, and officials can demand a bribe in addition of the license official fee. They tested the effect of this intervention during both single (one‐shot) and repeated interactions between citizens and officials. In their benchmark delivery setting, they gave officials monopoly power over a given location, so that citizens matched with an office had no choice but to pay the demanded bribe, if any. They then introduced bureaucratic competition by allowing citizens to move between the available offices on a map, after paying a small search cost, and obtain a license from any of the visited offices. Their results showed that allowing citizens to apply for a license from multiple offices lowers the size of bribes by more than 50%. “When officials are given the chance to periodically communicate with other competing officials before setting their bribe demands, the negative effect of competition on corruption is diminished. However, officials are unable to sustain collusion over time and, in the long‐run, communication among officials has no effect on bribe demands” (Ryvkin & Serra, 2017: 5).
28. After having demonstrated that the removal of bureaucrats’ monopoly power and the introduction of competition in service delivery significantly lower bribe demands and payments, Ryvkin & Serra, 2017,
  ^36^
  Ryvkin, D., & Serra, D. (2017). Is more competition always better? An experimental study of extortionary corruption (No. wp2015_10_01). Department of Economics, Florida State University.
   extended their previous work (Ryvkin & Serra, 2016) by testing whether, under competition, the number of offices providing the same service and the size of the costs that citizens have to sustain to visit a new office matter in the fight against extortionary corruption. In this laboratory experiment, they did not exogenously match each service recipient to a possibly corrupt service provider; but they allowed service recipients to search among multiple service providers and choose to receive a service or good from any of them. This framework allowed them to investigate whether changes in the number of available offices providing the same “license” affect the demand of bribes. Their results demonstrated that increasing competition among offices may reduce bribe demands only if it comes with a reduction in search costs. It is the reduction in search costs that is the driver of the reduction in bribes. The merely increase in the number of offices may actually lead to an increase in bribes demanded (if search cost are low) or to no change in corruption (if search costs are high). The joint findings of these two studies of Ryvkin and Serra suggested that while reducing monopoly by introducing competition in service delivery significantly reduces extortionary corruption, having a large number of bureaucrats providing the same service may increase bribe demands. Reducing citizens’ search costs when multiple offices exist (e.g., lowering transportation costs or through information sharing mechanisms) always reduces bribe demands and payments.

### Professional Identity

29. Falisse and Leszczynska (2015)
  ^37^
  Falisse, J. B., & Leszczynska, N. (2015). Professional Identity, Bribery and Public Service Delivery: Evidence from a Lab‐in‐the‐Field Experiment in Burundi (No. ECARES 2015‐07). ULB‐‐Universite Libre de Bruxelles.
   developed a lab‐in‐the‐field experiment with 527 public servants from Burundi who were asked to allocate rationed vouchers between anonymous citizens, some of whom tried to bribe them. The aim of their experiment was to test the effect of sensitization message that stressed professional identity and values of the public officials. Results showed that public servants exposed to these messages behaved in a fairer manner than those exposed to a standard anti‐corruption message or no message at all. In particular, they found evidence that reminding public servants of their professional identity and the qualities expected from it influences their delivery of the public service, i.e. vouchers allocation, but not their propensity to accept bribes. This type of intervention may be assimilated to policies that trigger the moral values when officials or citizens are confronted with a bribery temptation.
