# Supplementary material for: Public sector reforms and their impact on the level of corruption: A systematic review
Source: Campbell Syst Rev. 2021 May 24;17(2):e1173. doi: 10.1002/cl2.1173 (PMC8356278; doi:10.1002/cl2.1173)
Supplement: Supplementary file 1 — Supporting information [file CL2-17-e1173-s001.docx]

# Data and analyses

Analysis 1: Funnel plot for publication bias


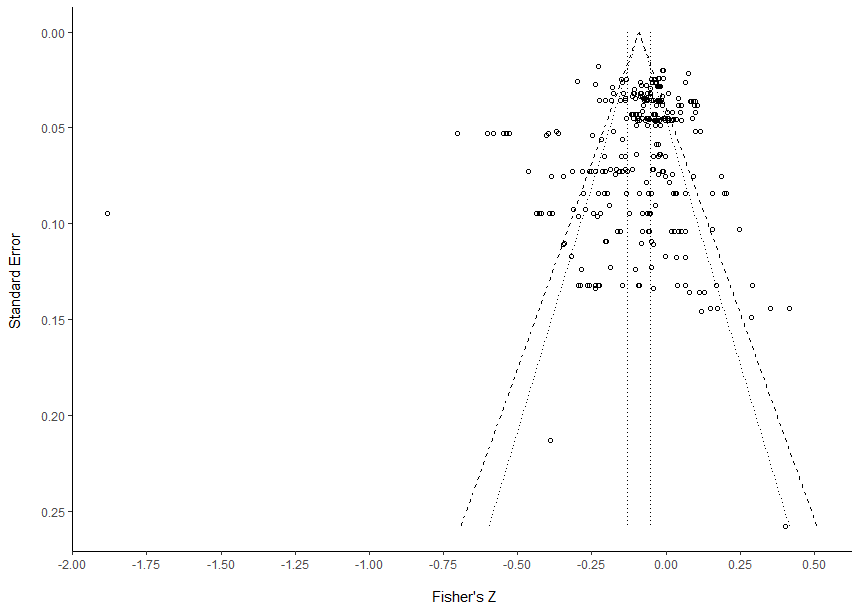


Analysis 2: t-statistics distribution


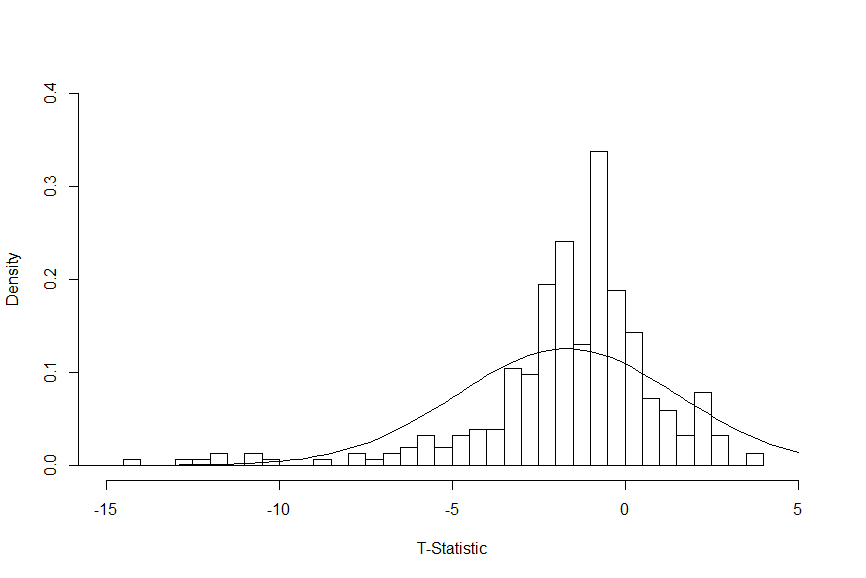


Analysis 3: Forest plot by type of intervention and corruption


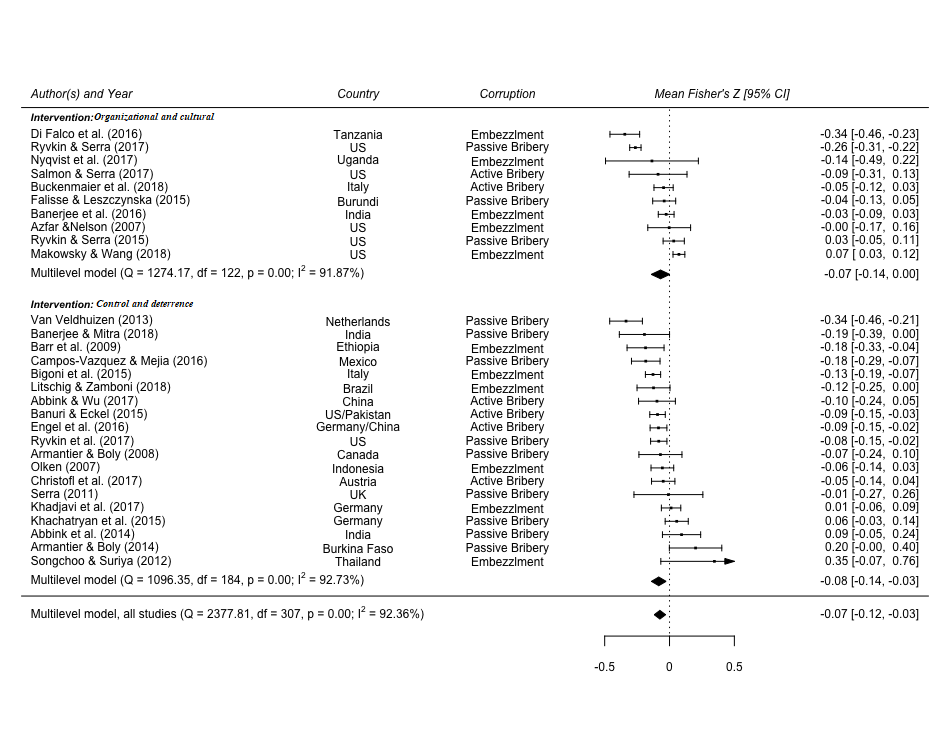


Analysis 4: Publication bias tests

|  |  | beta | se | T | ci (low) | ci (up) |
| --- | --- | --- | --- | --- | --- | --- |
| *Multiplicative* | Intercept | -0.038 | 0.029 | -1.316 | -0.094 | 0.019 |
|  | SE | -0.871 | 0.616 | -1.416 | -2.079 | 0.335 |
| *Additive* | Intercept | -0.073* | 0.033 | -2.180 | -0.142 | -0.004 |
|  | SE | -0.292 | 0.485 | -0.603 | -1.288 | 0.033 |

Multiplicative refers to a funnel plot asymmetry test using a multiplicative dispersion term – see Egger et al. (1997) and Stanley (2008). Additive refers to a funnel plot asymmetry test using an additive dispersion term – see Egger and Sterne (2005). Beta is the estimated coefficient; se its standard errors; t the t-statistic; ci (low) and ci (up) are, respectively, the lower and upper bounds of the confidence interval. Significance codes: p<0.001 '***' p<0.01 '**' p<0.05 '*'

Analysis 5: Results of the meta-analysis models

|  | beta | se | z | ci (low) | ci (up) |
| --- | --- | --- | --- | --- | --- |
| *RE* | -0.091 | 0.010 | -9.382 | -0.110 | -0.072 |
| *RE (cluster)* | -0.091 | 0.020 | -4.451 | -0.133 | -0.049 |
| *REs* | -0.074 | 0.023 | -3.290 | -0.118 | -0.030 |

Beta is the estimated coefficient; se its standard errors; z the z-statistic; ci (low) and ci (up) are, respectively, the lower and upper bounds of the confidence interval.

Analysis 6: Random effect Model, clustered standard error

| **Model results** | **beta** | **SE** | **z** | **p value** | **ci (low)** | **ci (up)** |
| --- | --- | --- | --- | --- | --- | --- |
| *Students vs other participants lab. Exp.* | -0.1007 **^.^** | 0.0492 | -2.0457 | 0.0557 | -0.2042 | 0.0027 |
| *Low.income* | -0.1496 * | 0.0604 | -2.4768 | 0.0234 | -0.2764 | -0.0227 |
| *Low.middle.income* | 0.0010 | 0.0396 | 0.0253 | 0.9801 | -0.0821 | 0.0841 |
| *Upper.middle.income* | -0.0840 **^.^** | 0.0480 | -1.7490 | 0.0973 | -0.1849 | 0.0169 |
| *Control/Deterrence vs Organizational/Cultural interventions* | 0.0034 | 0.0337 | 0.1016 | 0.9202 | -0.0674 | 0.0743 |
| *Economic vs other tyes interventions* | -0.0423 | 0.0369 | -1.1478 | 0.2661 | -0.1198 | 0.0351 |
| *More than one intervention* | -0.2386 ** | 0.0664 | -3.5934 | 0.0021 | -0.3781 | -0.0991 |
| *Embezzlement vs other types of corruption* | -0.0454 | 0.0324 | -1.4011 | 0.1782 | -0.1135 | 0.0227 |
| *Extortionary vs collusive corruption* | 0.0227 | 0.0277 | 0.8189 | 0.4236 | -0.0355 | 0.0809 |
| *Quality of the study* | -0.0041 **^.^** | 0.0022 | -1.8290 | 0.0840 | -0.0088 | 0.0006 |
| *Intercept* | 0.0737 | 0.0558 | 1.3211 | 0.2030 | -0.0435 | 0.1908 |

beta is the estimated coefficient; se its standard errors; z the z-statistic; p value the estimated p value. ci (low) and ci (up) are, respectively, the lower and upper bounds of the confidence interval. There are 308 effect sizes nested around 29 papers, with a mean of 10.62 effect sizes per study, a median of 8, a minimum of 2 and a maximum of 32. Significance codes: 0 '***' 0.001 '**' 0.01 '*' 0.05 '.' 0.1 ' ' 1.

Analysis 7: Multilevel Meta-Analysis Model

| **Model results** | **beta** | **SE** | **z** | **p value** | **ci (low)** | **ci (up)** |
| --- | --- | --- | --- | --- | --- | --- |
| *Students vs other participants lab. Exp.* | -0.0814 | 0.0944 | -0.8627 | 0.3883 | -0.2663 | 0.1035 |
| *Low.income* | -0.0669 | 0.0911 | -0.7342 | 0.4629 | -0.2455 | 0.1117 |
| *Low.middle.income* | 0.0828 | 0.0727 | 1.1402 | 0.2542 | -0.0596 | 0.2253 |
| *Upper.middle.income* | 0.0390 | 0.0716 | 0.5449 | 0.5858 | -0.1013 | 0.1793 |
| *Control/Deterrence vs Organizational/Cultural interventions* | -0.1131 ****** | 0.0425 | -2.6611 | 0.0078 | -0.1965 | -0.0298 |
| *Economic vs other types interventions* | -0.0098 | 0.0390 | -0.2514 | 0.8015 | -0.0862 | 0.0666 |
| *More than one intervention* | -0.1337 ***** | 0.0522 | -2.5592 | 0.0105 | -0.2360 | -0.0313 |
| *Embezzlement vs other types of corruption* | -0.1084 ***** | 0.0476 | -2.2750 | 0.0229 | -0.2018 | -0.0150 |
| *Extortionary vs collusive corruption* | 0.0165 | 0.0657 | 0.2512 | 0.8017 | -0.1123 | 0.1453 |
| *Quality of the study* | -0.0030 | 0.0062 | -0.4771 | 0.6333 | -0.0152 | 0.0093 |
| *Intercept* | 0.1088 | 0.1073 | 1.0146 | 0.3103 | -0.1014 | 0.3191 |

beta is the estimated coefficient; se its standard errors; z the z-statistic; p value the estimated p value. ci (low) and ci (up) are, respectively, the lower and upper bounds of the confidence interval. There are 308 effect sizes nested around 29 papers, with a mean of 10.62 effect sizes per study, a median of 8, a minimum of 2 and a maximum of 32. Significance codes: 0 '***' 0.001 '**' 0.01 '*' 0.05 '.' 0.1 ' ' 1.

Analysis 9 - Multilevel Meta-Analysis Model: laboratory-based studies only

| **Model results** | **beta** | **SE** | **z** | **p value** | **ci (low)** | **ci (up)** |
| --- | --- | --- | --- | --- | --- | --- |
| *Students vs other participants lab. Exp.* | -0.2532 | 0.1344 | -1.8831 | 0.0597 | -0.5167 | 0.0103 |
| *Low.income* | -0.2 | 0.1067 | -1.8751 | 0.0608 | -0.409 | 0.009 |
| *Low.middle.income* | 0.1036 | 0.0889 | 1.1654 | 0.2439 | -0.0707 | 0.2779 |
| *Upper.middle.income* | 0.0817 | 0.0857 | 0.9537 | 0.3402 | -0.0863 | 0.2497 |
| *Control/Deterrence vs Organizational/Cultural interventions* | -0.0978* | 0.048 | -2.0381 | 0.0415 | -0.1918 | -0.0038 |
| *Economic vs other types interventions* | -0.0165 | 0.0453 | -0.3652 | 0.715 | -0.1053 | 0.0722 |
| *More than one intervention* | -0.1461* | 0.0599 | -2.4382 | 0.0148 | -0.2635 | -0.0287 |
| *Embezzlement vs other types of corruption* | 0.0545 | 0.0846 | 0.6441 | 0.5195 | -0.1113 | 0.2202 |
| *Extortionary vs collusive corruption* | 0.0312 | 0.0869 | 0.3586 | 0.7199 | -0.1391 | 0.2014 |
| *Quality of the study* | -0.002 | 0.0128 | -0.1558 | 0.8762 | -0.027 | 0.0231 |
| *Intercept* | -0.002 | 0.0128 | -0.1558 | 0.8762 | -0.027 | 0.0231 |

beta is the estimated coefficient; se its standard errors; z the z-statistic; p value the estimated p value. ci (low) and ci (up) are, respectively, the lower and upper bounds of the confidence interval. There are 250 effect sizes nested around 25 papers. Significance codes: p<0.001 '***' p<0.01 '**' p<0.05 '*'.

Analysis 10: Random effect Model Coefficients: field-based studies only (k = 58; tau^2 estimator: REML)

|  | *estimate* | *ci (low)* | *ci (up)* |
| --- | --- | --- | --- |
| tau^2 (estimated amount of total heterogeneity) | 0.000 (se = 0.000) | 0.000 | 0.003 |
| tau (square root of estimated tau^2 value) | 0.016 | 0.000 | 0.052 |
| I^2 (total heterogeneity / total variability) | 14.267% | 0.000 | 64.887 |
| H^2 (total variability / sampling variability): | 1.166 | 1.000 | 2.848 |

Test for Heterogeneity: Q(df = 57) = 76.273, p-val = 0.045

Analysis 11: Random effect Model Results: field-based studies only

| *beta* | *se* | *z* | *ci (low)* | *ci (up)* |
| --- | --- | --- | --- | --- |
| -0.053*** | 0.006 | -9.489 | -0.064 | -0.042 |

beta is the estimated coefficient; se its standard errors; z the z-statistic; ci (low) and ci (up) are, respectively, the lower and upper bounds of the confidence interval. There are 58 effect sizes nested around 4 papers. Significance codes: p<0.0001 '***'

The random-effects model on field-experiments only confirms the negative effect of interventions on corruption. However, it suffers from the following drawbacks: a) the between-effect sizes heterogeneity estimate is unreliable (i.e., I^2 ranges from 0% to 65%); b) the overall heterogeneity of the data is (falsely) estimated to be 0 (tau^2 =0) also due to the wide differences in the number of points of effect sizes of the various studies (one paper contributes with only two estimates, while the other three respectively provide 24, 18 and 12 estimates); c) SEs and confidence intervals are wrongly estimated because they refer to 58 estimate points considered independently. SEs should be clustered (as we did for the overall model) but it is not possible in this case due to the low number of groups (only four papers).

# Online supplements

List of online supplements

1. List of search strings and related results per database
2. Data extraction of included studies
3. Assessment of risk of bias of included studies
4. Assessment of journals
